# Epigenetic Mechanisms of Endocrine-Disrupting Chemicals in Breast Cancer and Their Impact on Dietary Intake

**DOI:** 10.3390/jox15010001

**Published:** 2024-12-24

**Authors:** Desh Deepak Singh

**Affiliations:** Amity Institute of Biotechnology, Amity University Rajasthan, Jaipur 303002, India; ddsbms@gmail.com; Tel.: +91-9450078260

**Keywords:** endocrine disruptors, epigenetic, breast cancer, dietary exposure

## Abstract

Addressing the consequences of exposure to endocrine-disrupting chemicals (EDCs) demands thorough research and elucidation of the mechanism by which EDCs negatively impact women and lead to breast cancer (BC). Endocrine disruptors can affect major pathways through various means, including histone modifications, the erroneous expression of microRNA (miRNA), DNA methylation, and epigenetic modifications. However, it is still uncertain if the epigenetic modifications triggered by EDCs can help predict negative outcomes. Consequently, it is important to understand how different endocrine disrupters or signals interact with epigenetic modifications and regulate signalling mechanisms. This study proposes that the epigenome may be negatively impacted by several EDCs, such as cadmium, arsenic, lead, bisphenol A, phthalates, polychlorinated biphenyls and parabens, organochlorine, and dioxins. Further, this study also examines the impact of EDCs on lifestyle variables. In breast cancer research, it is essential to consider the potential impacts of EDC exposure and comprehend how EDCs function in tissues.

## 1. Introduction

Breast cancer is a significant public health issue worldwide. According to recent World Health Organization (WHO) data, breast cancer caused 670,000 deaths globally in 2022 [1]. Roughly half of all breast cancers occur in women with no specific risk factors other than sex and age. In 2022, breast cancer was the most common cancer in women in 157 out of 185 countries. Breast cancer occurs in every country in the world [1]. Approximately 0.5–1% of breast cancers occur in men, highlighting the importance of awareness and education regarding this disease for all genders [1,2]. Early detection through regular screenings can significantly improve survival rates, making it crucial for individuals to understand their risk [1,2]. Endocrine-disrupting chemicals (EDCs) are defined broadly as exogenous molecules that interfere with the endogenous hormonal axis at any level [3,4]. This encompasses the synthesis, metabolism, transport, and administration of hormones; disruptions to hormone receptor expression and downstream signals; the activation or inhibition of hormonal signals; and changes to epigenetic regulation [3,4]. EDCs include both natural molecules that affect estrogen signalling, such as phytoestrogens (e.g., genistein, which is prevalent in soy), and synthetic chemicals intended for therapeutic purposes, such as those used as adjuvant therapies in breast cancer [5,6]. EDCs have diverse origins, structures, and actions; it is believed that EDCs predominantly function through nuclear hormone receptors, such as estrogen receptors (ERs) and progesterone receptors (PRs) [7,8]. There is increasing evidence indicating that EDCs alter the epigenetic landscape in common diseases, including cardiovascular, pulmonary, and neurological conditions, as well as malignancies, particularly breast cancer [2,3]. Hormones operate as ligands for nuclear receptors, which can influence gene expression directly by binding to DNA or indirectly through transcription factors [4,7]. Hormones can also signal through non-genomic routes by engaging with membrane nuclear receptors or cytoplasmic receptors, triggering signalling cascades via secondary messengers (SMs) that produce rapid physiological effects without affecting gene expression [8.9]. Endocrine-disrupting substances can bind to receptors and imitate endogenous hormones, but they also can influence hormone signalling in a variety of other ways [9]. EDCs can interact with various receptors, including non-nuclear receptors, either as agonists, facilitating genomic connections, or as antagonists, causing a conformational change in the receptor to prevent activation [8,9,10,11]. Importantly, EDCs can disrupt endogenous hormone synthesis and breakdown, altering hormone levels. Recent research has also shown that EDCs can affect genome methylation and histone alterations through an epigenetic mechanism [6,7,8,9,10].

**Figure 1:**
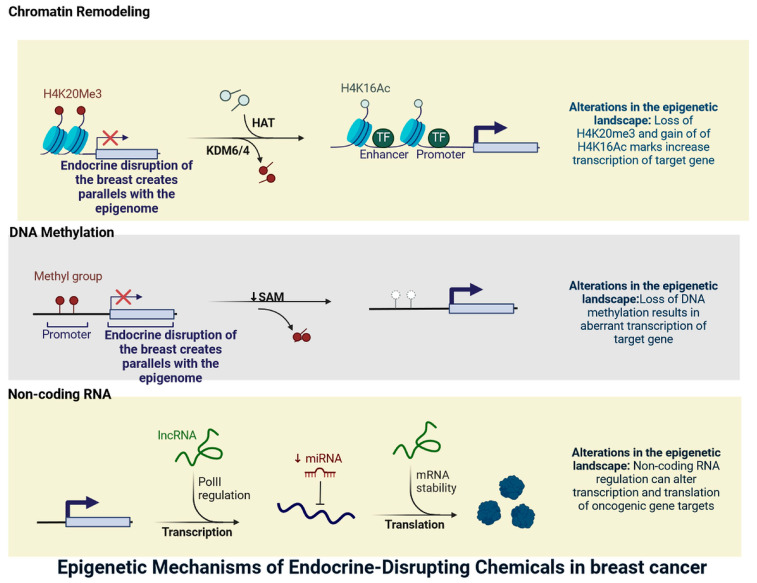
The effects of endocrine-disrupting chemicals (EDCs) and the mechanism(s) by which epigenetic modification, including DNA methylation, expression of aberrant microRNA (miRNA), and histone modification, is one mechanism assumed to be a primary pathway leading to the untoward effects of endocrine disruptors (the figure was designed using BioRender graphics: https://www.biorender.com, accessed on 28 July 2024).

**Figure 2:**
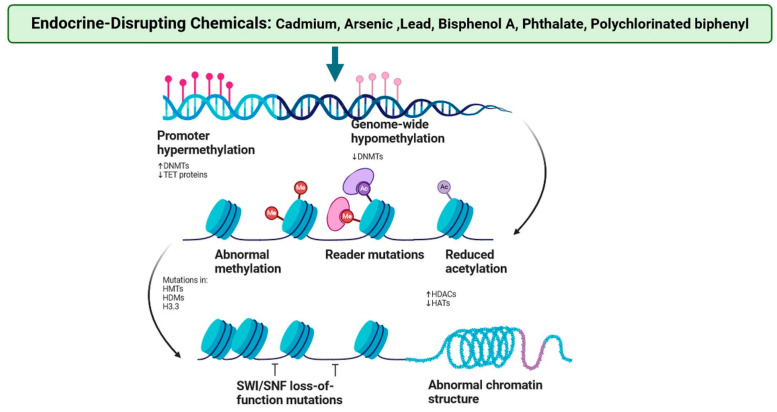
Endocrine disruptors and risk factors mediate epigenome modifications that increase the risk of breast cancer. EDC may prolong puberty and increase mammary epithelial cell proliferation, allowing for a longer duration or faster rate of epigenetic remodelling of the developing mammary gland, resulting in chromatin destabilisation, mispackaging of genes in active/inactive domains, and aberrant expression of genes in key regulatory pathways. Mutations in HMTs (histone methyltransferases), HDMs (histone methyltransferases), and H3.3 (Histone variant H3.3) increase Histone deacetylase 1 (HDAC1) but reduce HATs (histone acetyltransferases) (the figure was designed using BioRender graphics: https://www.biorender.com, accessed on 28 July 2024).

Epigenetic modifications encompass an array of DNA and histone modifications that affect levels of gene expression without changing the underlying coding sequence [3,4]. Histone alterations can control the recruitment of DNA Methyltransferases (DNMTs) to CpG (cytosine and guanine dinucleotide) sites, whereas methylated CpG sites promote the recruitment of methyl-binding proteins to the genomic area; in turn, these proteins recruit histone deacetylase enzymes [10,11,12]. These enzymes facilitate the removal of acetyl groups from histones, leading to a more compact chromatin structure and transcriptional repression. Consequently, the interplay between histone modifications and DNA methylation plays a crucial role in governing gene expression and maintaining cellular identity (Figure 1 and Figure 2) [2,3,4,5,6,7,8,9,10,11,12,13,14,15]. The degradation of histone tail lysine residues causes the local chromatin structure to constrict, preventing access to transcriptional machinery and thereby repressing gene transcription [3,5,6]. Small non-coding RNAs (ncRNAs) also serve as a key element of the cell’s epigenetic processes of regulation. The ncRNAs discovered in mammalian systems include microRNA (miRNA), endogenous siRNA, and PIWI-interacting miRNA (piRNA) [6,7]. They vary in their genetic origin and post-translational processing, but they all have the same function: the post-transcriptional down regulation of the expression of target genes as well as other non-coding RNAs [7,8]. Long non-coding RNAs can be generated from gene regulatory areas or mitochondrial DNA. They mostly impact the local genetic area of their origin, acquiring transcription factors or other epigenetic modifications and developing breast cancer (Figure 1 and Figure 2) [2,3,4,5,6,7,8,9,10,11,12,13,14,15].

Environmental epigenetic changes offer clear molecular pathways through which variables or hazardous substances may influence a genetic cascade of occurrences that contribute to the development of breast cancer [14,15,16,17]. There are crucial windows of sensitivity in which these components alter and affect vital phases of development. A tumour sample panel study showed that increased DNA methylation is an indicator of ER-positive tumours that are involved in breast cancer [3,14,15,16,17,18,19]. The identification of epigenetic markers modified by EDCs in the environment will thus provide a potential method of early detection, predictive value, and functional information about the chemical itself [18,19,20,21,22]. The WHO and IARC (International Agency for Research on Cancer) have identified EDCs that are also found in food. These substances fall into several categories, including phthalates, organochlorine insecticides, polychlorinated biphenyl, polybrominated diphenyl ethers, dioxins, bisphenol A, and heavy metals [22,23]. This study focuses on EDCs that may alter the epigenome and mainly affect endocrine-responsive organs such as the breast, addressing the effects of food intake and lifestyle factors on health by investigating the effects of various environmental factors on gene expression and highlighting the potential long-term consequences of exposure to these endocrine-disrupting chemicals. Data searches were conducted using Google, PubMed, Scopus, Google Scholar, and Web of Science. This study emphasises the need for further research to understand the mechanisms through which EDCs influence epigenetic modifications and their implications for human health.

## 2. Endocrine Disruption in Breast Cancer Parallels the Epigenome

Environmental and lifestyle factors are considered some of the major influencing components that increase breast cancer risk [12,13,24,25]. EDCs may epigenetically alter immune cell differentiation, creating an immunosuppressive environment through the increased differentiation of regulatory T cells (Tregs), which impair anti-tumour immunity [22,23,24]. EDC-induced epigenetic alterations might cause aberrant cytokine production, establishing a pro-tumour milieu by increasing anti-inflammatory cytokines (e.g., IL-10) and decreasing pro-inflammatory cytokines (e.g., IFN-γ) [25]. EDCs can cause epigenetic alterations in stromal cells, such as cancer-associated fibroblasts (CAFs) and endothelial cells. This causes increased release of growth factors (e.g., VEGF) that promote angiogenesis [26]. Changes in metalloproteinase expression cause altered extracellular matrix (ECM) remodeling [22]. EDCs can epigenetically upregulate immune checkpoint molecules such as PD-L1 on tumour cells, allowing them to avoid immune detection [23]. EDCs may activate NF-κB, causing low-grade inflammation and tumour growth [22]. EDC-induced epigenetic alterations in immune cells (such as T cells and macrophages) can enhance their suppressive effects, allowing the TME to avoid immunological responses [22]. EDCs can epigenetically remodel stromal and immune cells, resulting in niches that promote tumour cell survival, invasion, and metastasis [22,23,24,25,26]. The link between high amounts of EDCs in individuals and a higher probability of breast cancer has been frequently investigated, with no conclusive evidence demonstrating a relationship [21,22,23,24]. An EDC can act as an estrogen mimic and may interact with the ligand-binding region of ER-α, increasing cellular proliferation, possibly by slowing apoptosis, and producing a gene expression profile connected with a poor cancer survival prognosis. This has led to enhanced growth of mammary glands in rats, demonstrating the effects of processing and storing EDCs instead of exposure via maternal blood, where circulation levels were undetermined [8,9,25,26,27,28,29,30,31,32]. This section discusses how cell signalling mechanisms and exposure to common EDCs might impact epigenetic changes and the risk of breast cancer. EDC-induced epigenetic alterations can inhibit genes involved in immune cell activation and cytotoxicity, reducing the immune system’s ability to attack tumour cells. The epigenetic effects regulated by EDCs in breast cancer are summarised in Table 1.

### 2.1. Cadmium

**Figure 3:**
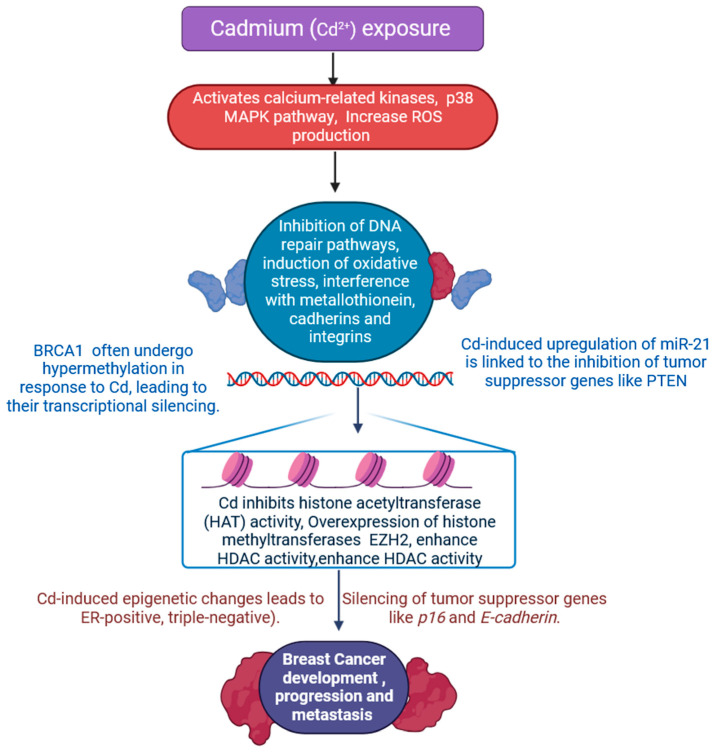
Epigenetic control is crucial in mitigating the toxic effects of cadmium (Cd^2+^), a heavy metal that causes serious health and environmental problems. Cadmium can alter cellular homeostasis and cause cancer, often through non-genetic mechanisms such as DNA methylation, histone changes, and microRNA (miRNA) control. The disrupting effects of cadmium on MAPK pathways on cellular signalling and health. Figure highlights the direct and indirect effects of Cd^2+^ interference on cellular function, which lead to aberrant cell responses and elevated breast cancer. These disruptions can result in altered gene expression and impaired cell proliferation, ultimately contributing to tumourigenesis (the figure was designed using BioRender graphics: https://www.biorender.com, accessed on 8 August 2024).

Cadmium (Cd^2+^) is a metal that exists as a natural component of the Earth’s crust [44]. Cd^2+^ is a heavy metal that has been linked to breast cancer and is known to cause epigenetic modifications in breast cancer cells, potentially influencing gene expression and contributing to tumour progression [44,45]. Research continues to explore the mechanisms through which cadmium affects cellular pathways, aiming to identify potential interventions or preventative measures for those at risk [46,47]. Understanding the specific pathways influenced by cadmium exposure may lead to targeted therapies that could mitigate its harmful effects [48,49,50]. Cd^2+^ has been demonstrated to cause a variety of epigenetic alterations. These alterations may cause modifications in gene expression, as observed both in vitro and in vivo. Cd^2+^ affects key signalling pathways, either directly or indirectly, including the NF-κB, p53, and MAPK pathways (Figure 3) [44,45,46,47,48,49,50,51,52,53,54]. The modification enhances the pathological situation, influences the functions of cells, and triggers unique biological responses in various types of cells, enabling them to react to a variety of external signals, including growth hormones, cellular stress, and inflammatory stimuli [50,51,52,53,54]. These responses are crucial for maintaining homeostasis and adapting to changing environments. By modulating cellular activities, the modification plays a significant role in processes such as tissue repair and immune response [54]. The Ras-Raf-MEK-ERK signalling pathway is activated by external inputs, and under normal conditions, it stimulates ERK and the expression of specific genes required for regular cell growth and division, maintaining the physiological state and functionality of cells [54]. However, the dysregulation of this pathway can lead to uncontrolled cell proliferation and survival, contributing to the development of various cancers [46,47,48,49,50]. Understanding the intricacies of the Ras-Raf-MEK-ERK signalling pathway is crucial for developing targeted therapies aimed at restoring normal cellular functions (Figure 3) [50,51,52,53,54,55,56,57,58,59,60]. The prolonged stimulation of the ERK pathway can occur from chronic Cd^2+^ toxicity [54,55], leading to altered signal transduction and clinical consequences in different cells since importunate ERK activation increases cell proliferation, suppresses mechanisms for programmed cell death, and tampers with cell cycle control [56,57,58]. Cd^2+^ activates this process, triggering a variety of cellular reactions such as transcription factors controlling the cell cycle, apoptosis, proliferation, and the regulation of genes essential for cell survival [55,56,57]. The active route helps cells adapt to or fend off the harmful effects of Cd^2+^ by encouraging the phosphorylation of the transcription factor c-Jun, an essential stage in cellular stress responses [50,51,52]. High-dose or increased consumption of Cd^2+^ can result in excessive JNK pathway activation, which can impede cellular functions such as autophagy, apoptosis, DNA damage, and cytoskeletal instability [56,57,58,59,60]. These modifications can eventually lead to cell problems and pathogenic alterations, promoting the development of diseases like breast cancer [59,60]. In the final phase, they impact long-term survival by modulating responses to inflammation, apoptosis, and survival signals [48,49,50,51,52]. Comparable to activating the JNK pathway, Cd^2+^ directly interacts with specific cell surface receptors or channels, such as calcium channels, to cause signalling downstream that alters intracellular Cd^2+^ concentrations [58,59,60,61,62]. This, in turn, indirectly activates a number of calcium-related kinases, which activate the p38 MAPK pathway. An increase in ROS brought on by Cd^2+^ either directly or indirectly activates the p38 MAPK pathway through upstream kinases such as apoptosis signal-regulating kinase 1 (ASK1), enabling an oxidative stress response that helps cells resist the harmful effects of Cd^2+^ (Figure 3) [50,51,52,53,54,55,56,57,58,59,60]. One important signalling mechanism that regulates where cells react to inflammatory and immune responses is the NF-κB pathway; NF-κB is usually linked to the inhibitory protein IκB and persists in the cytoplasm in a state of inactivity [60,61,62,63,64]. Cell survival signals trigger inflammatory processes, and Cd^2+^ may stimulate the NF-κB pathway, thus mediating cellular responses to its toxicity. Based on the result of a few studies, Cd^2+^ enhances the response to inflammation by increasing NF-κB activation via an ROS-dependent mechanism [65,66,67]. The two cell surface receptors that Cd^2+^ can mimic or activate are receptor-interacting serine/threonine protein kinase 1 (RIP1) and receptor-associated factor 6 (TRAF6). As a result, signalling molecules and related adaptor proteins become activated, which then increases IKK activity and opens the NF-κB pathway [63,64,65,66,67]. Cell growth and development, differentiation, apoptosis, and responses to stress are all significantly influenced by the complex and wide-ranging biological impacts that can arise from Cd^2+^ disruption of cellular signalling networks [62,63,64,65,66].

### 2.2. Arsenic

**Figure 4:**
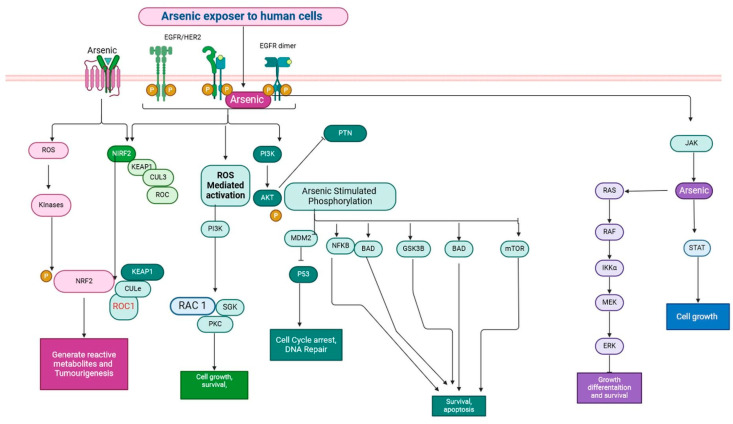
Growth factor receptors activated by arsenic stimulate the PI3K/AKT pathway, which promotes angiogenesis, cell cycle progression, and cellular proliferation. Through the TRAIL receptor and reactive oxygen species, arsenic triggered apoptosis by upregulating pro-apoptotic markers and down-regulating anti-apoptotic signs. These mechanisms illustrate how arsenic can exert both pro-survival and pro-death signals within cells, leading to complex interplay in tumour standing these pathways is crucial for developing targeted therapies that could mitigate the adverse effects of arsenic exposure while potentially harnessing its apoptotic capabilities against cancer cell biology. Understanding these pathways is crucial for developing targeted therapies that could mitigate the adverse effects of arsenic exposure while potentially harnessing its apoptotic capabilities against cancer cells (the figure was designed using BioRender graphics: https://www.biorender.com, accessed on 9 August 2024).

Arsenic (As) is a hazardous element present in the Earth’s crust. It is commonly utilised in lead alloys (batteries and weapons) and semiconductor circuits [68,69]. Arsenic trioxide was used to make herbicides and cure wood products, but both applications have been mostly discontinued since its toxicity was identified and associated with mutation, genotoxicity, and breast cancer [69]. Chronic arsenic exposure can activate oncogenes or tumour suppressors owing to arsenic’s impact on miRNA, contributing to the overexpression of hypoxia factors, and it has been linked to lung, skin, bladder, and other cancers [70,71]. MiR-182-5p is suppressed, and the overexpression of these factors may increase arsenic’s carcinogenic potential [72,73,74]. Several genes are expressed via cellular proliferation, differentiation, transformation, and apoptosis with the aid of mitogen-activated protein kinase [72,73]. The three most significant members of this family are p38, extracellular signal-regulated kinases (ERKs), and c Jun N-terminal kinase (JNK) [73,74,75,76]. ERK and p38 signalling are activated by Ras/Raf/Mek signalling; however, JNK activation is mediated by Rac, Rho, and MEKK 1-4. Arsenic poisoning occurs due to the MAPK cascade [70,71,72,73,74]. Exposure to arsenic has been associated with the phosphorylation of the epidermal growth factor receptor (EGFR), which then triggers cellular proliferation, tumour invasion, and angiogenesis (Figure 4) [68,69,70,71,72,73,74,75]. The transmembrane tyrosine kinase receptor known as EGFR, or epithelial growth factor receptor, is a member of the ErB family and is found on the surface of epithelial cells [75,76]. In in vivo settings, transcription is triggered by EGFR’s continued attachment to the AT-rich consensus region in the promoter of cyclin D1, and the Ras/MAP kinase pathway can also be activated by EGF [77,78]. The overexpression of cyclin D1 will trigger the development of the cyclin D1-cdk4 complex, which, in turn, will promote the transcription factors E2F and cyclin via the phosphorylation of pRB, a growth suppressor, finally resulting in the progression of the cell cycle [76,77,78,79,80]. Recent experiments using p53 antisense oligonucleotide-induced p53 suppression in human gastric cancer and human glioblastoma cell lines showed suppressed apoptosis and no caspase activation [81,82]. This result demonstrates that p53 plays a role in apoptosis caused by arsenic [81,82]. MMPs have a wide range of physiological and pathological functions, participating in angiogenesis, tissue remodelling, trophoblastic implantation, tumour development, wound healing, and degenerative illnesses of various kinds, among others [83,84,85,86,87,88,89]. The majority of MMPs are secreted by cells in a proactive state, and they are crucial regulators of a variety of pathophysiological processes when they are activated [87,88]. The over-secretion of pro-inflammatory and growth-promoting cytokines may be induced by NF-kβ and AP-1, which might lead to the onset of carcinogenesis [89]. Arsenate’s effects on telomeres and telomerase, as well as cell proliferation and death, have been evaluated in HL-60 and HaCaT cells in vitro [68]. Low doses (0.1–1 µM) of arsenate increased telomerase activity, extended telomeres, and encouraged cell proliferation. High doses (>1–40 µM) of arsenide reduced telomerase activity, shortened telomeres, and caused apoptosis [68,69]. The results of research comparing cell lines with and without telomerase activity indicated that telomerase was implicated in arsenic-induced apoptosis [68]. These findings show that arsenic’s carcinogenic effects may be partly due to an increase in telomerase activity, which promotes cell proliferation, as well as its anticancer effects, which exert oxidative stress and cause telomeric DNA attrition and death [69]. These investigations suggest that arsenic plays an important role in the development of breast cancer.

### 2.3. Lead

**Figure 5:**
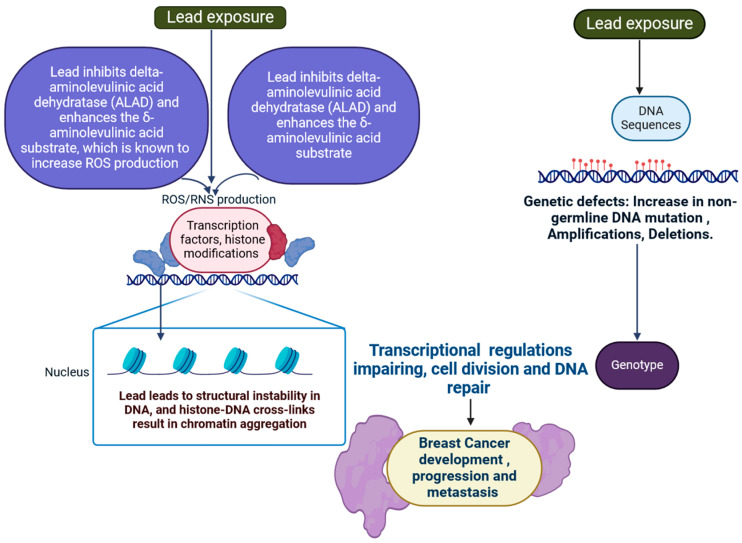
Lead inhibits delta-aminolevulinic acid dehydratase (ALAD) and enhances the δ-aminolevulinic acid substrate, which is known to increase ROS production and oxidative stress within cells. Epigenetic alterations can modify gene expression without changing the underlying DNA sequence, making them an important role in breast cancer growth and progression (the figure was designed using BioRender graphics: https://www.biorender.com, accessed on 10 August 2024).

The outermost layer of the planet is composed of lead (Pb) [90]. Lead emissions from metal smelters and mines are significant sources of environmental pollution, impacting both air and water quality [91]. Long-term lead exposure can lead to serious health issues in humans and wildlife, necessitating stringent regulations and monitoring efforts [92]. Many medical conditions, such as neurological, haematological, reproductive, and gastrointestinal illnesses, are the result of exposure to lead [92,93,94,95,96,97]. Exposure to lead changes cell regulatory mechanisms and influences genetic and epigenetic regulations. Epigenetic alterations can modify gene expression without changing the underlying DNA sequence, making them important in breast cancer growth and progression [98,99,100]. The expression of genes that encode certain miRNAs is one way that Pb affects the epigenetic regulation of genes throughout breast cancer development [100,101,102]. Lead inhibits delta-aminolevulinic acid dehydratase (ALAD) and enhances the δ-aminolevulinic acid substrate, which is known to increase ROS production [103,104,105]. Two specific types of enzymes are inhibited by lead: ALAD and glutathione reductase (GR). Investigations have indicated that lead disrupts the cycle that decreases glutathione levels by converting oxidised glutathione (GSSG) into reduced glutathione (GSH) [105]. Lead leads to structural instability in DNA, and histone–DNA crosslinks result in chromatin aggregation [104,105]. It has been discovered that lead acts as a mitogen to promote liver cell proliferation in vivo [104,105,106]. Biological impairment may result from the harmful effects of free ROS and RNS when there is an excess of ROS/RNS production or an antioxidant deficiency. In light of this, MDA is often employed as a lipid peroxidation biomarker. The aforementioned data support the hypothesis that reactive oxygen species (ROS), markers of oxidative stress, are produced by lead (Figure 5) [93,94,95,96,97,98,99,100,101,102,103,104,105,106]. Among them, ROS/RNS signalling transduction normally aids in proliferation, differentiation, and transformation in a time- and dosage-dependent manner, whereas ROS/RNS are frequently activated by responses to stress and are involved in apoptosis and proliferation arrest (Figure 5) [93,94,95,96,97,98,99,100,101,102,103,104,105,106]. Understanding the interaction of lead exposure and epigenetic changes could provide important insights into breast cancer prevention and treatment options.

### 2.4. Bisphenol A

**Figure 6:**
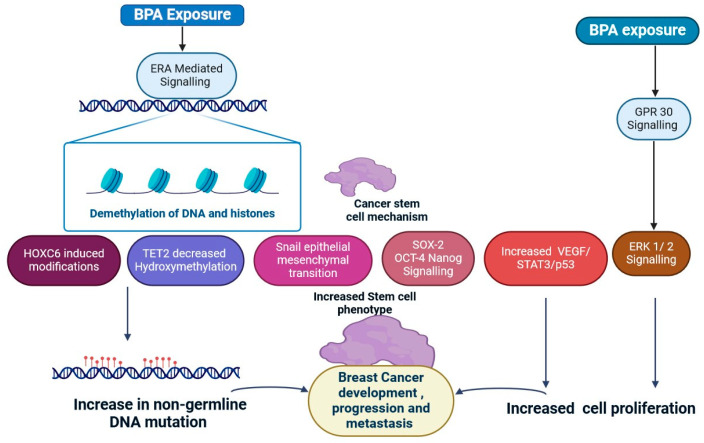
Potential pathways underlying BPA-induced breast cancer formation and progression. Bisphenol A (BPA), oestrogen receptor alpha (ERA), G-protein-coupled receptor 30 (GPR30), ten-eleven translocation 2 (TET2), Snail family zinc finger protein (SNAIL), and extracellular signal-regulated kinase 1/2 (ERK1/2). These elements interact in intricate ways to alter biological pathways, eventually leading to cancer linked with BPA exposure. Understanding these pathways is critical for developing tailored treatments and prevention methods for BPA-induced breast cancer (the figure was designed using BioRender graphics: https://www.biorender.com, accessed on 12 August 2024).

Bisphenol A (BPA) is one of the most extensively researched endocrine disruptors because it is readily converted into nonbioactive metabolites [107]. BPA mimics estrogen, causing breast cancer cells to proliferate. BPA can interact with estrogen receptors in cells, causing alterations in proliferation, apoptosis, and migration [108]. BPA can alter the environment surrounding a tumour, which is critical for cancer growth, and can induce hypermethylation, an epigenetic process that increases the risk of breast cancer [109]; for example, BPA can cause hypermethylation of BRCA1 in human mammary epithelial cells [109,110]. It was also demonstrated that exposure to BPA causes hypermethylation of the carnitine palmitoyl transferase 1A (CPTA1) gene [111]. This enzyme facilitates the entry of long-chain fatty acids into mitochondria and stimulates their oxidation by transferring acyl groups from fatty acids from coenzyme A to carnitine [109,110,111,112]. It is interesting to consider that BPA has been demonstrated to reduce DNA methylation via coat colour and enzymes like PDE type 4 variations [112,113]. This may initiate signalling pathways that lead to breast cancer and encourage malignancy. The inappropriate activation of oestrogen signalling is a significant contributing factor to the development of BC [111,112,113,114]. The activation of AKT and ERK1/2 is necessary for the proliferative and prosurvival effects of BPA in TNBC cells [111,112,113,114,115]. BPA elevated the expression of mRNA and proteins linked to migration in TNBC cells, including matrix metalloproteinase-2 (MMP2) [116,117]. Members of the nuclear receptor superfamily, or ERRs, are commonly referred to as orphan receptors since they do not have endogenous ligands. BPA stimulates the expression of EERγ in oestrogen-receptor-positive (ER+) breast cancer cells via phosphorylating ERK1/2 [117,118]. These results demonstrated that BC cell proliferation is stimulated by the EERγ/ERK1/2 axis [58,59]. Bisphenol A (BPA), oestrogen receptor alpha (ERA), G-protein-coupled receptor 30 (GPR30), ten-eleven translocation 2 (TET2), SNAIL, and extracellular signal-regulated kinase 1/2 (ERK1/2). Some studies have analysed the role of epigenetic modifications caused by BPA, particularly in relation to genes like TET2, in breast cancer development (Figure 6) [112,113,114,115,116,117,118]. BPA exposure can cause changes in gene expression while leaving the underlying DNA sequence unchanged [119]. These changes, which include DNA methylation and histone modification, can impair normal cellular functioning and promote neoplastic pathways, ultimately leading to the development and spread of breast cancer (Figure 6) [112,113,114,115,116,117,118]. Research has demonstrated that an increase in HOXB9 in BC is associated with neovascularisation, tumour invasion, and disease progression. The HOXB9 gene promoter contains a putative oestrogen response element (ERE4) that controls how the protein reacts to BPA and E2 [116]. BPA is sensitive to mammotrophic hormones and increases the risk of breast cancer in later life [116]. Research supporting this notion showed that BPA altered the expression and location of type 1 BMP receptors, reduced the development of BMPs in mammary fibroblasts, and impacted stem cells’ sensitivity to BMP signalling [116,117,118]. By activating SMAD1/5/8 phosphorylation, BPA enhanced BMP signalling [116]. Recent investigations suggest that BPA may activate signalling pathways that are linked to cancer, resulting in DNA damage, stem cell differentiation, and epigenetic changes [111,112,113,114,115,116,117,118,119].

### 2.5. Phthalates

**Figure 7:**
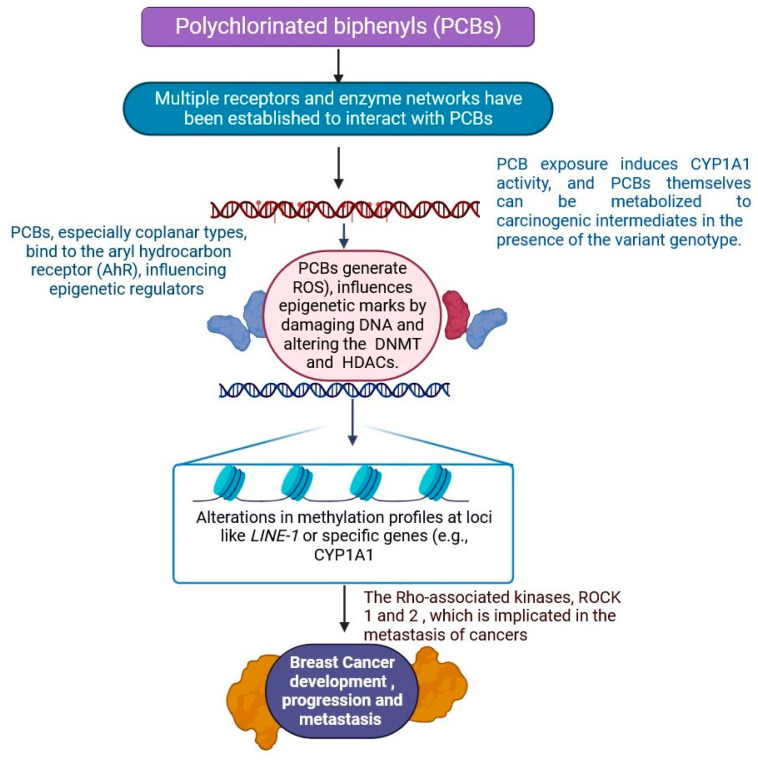
All the way through the Plyc, Mekk, IRAK, and PLC-β signalling pathways, phthalates altered the gene expression in breast cancer. These changes in gene expression could potentially influence tumour growth and metastasis, highlighting the need for further research into the mechanisms by which phthalates affect cellular processes. Understanding these pathways may lead to new therapeutic strategies for breast cancer treatment (the figure was designed using BioRender graphics: https://www.biorender.com, accessed on 13 August 2024).

Toys, nipples, wall coverings, floor tiles, pacifiers, teethers, textiles, vinyl floors, beds, household goods, and medical equipment are all made with phthalates, which are liquid plasticising agents [119]. Phthalates have the potential to disrupt epigenetic processes that are critical for gene expression development and maintenance [120]. Epigenetic changes can activate or silence genes, resulting in pathogenic conditions [121]. Phthalates can mimic hormones, potentially affecting the body’s estrogen, progesterone, and androgen systems [121]. Phthalates are frequently found in human urine, serum, and milk because they are not chemically bonded to plastic, making them easier to absorb [122]. Urine concentrations of phthalates are linked to many CpG sites that exhibit variable methylation [123,124,125]. In skeletal muscle cells, the Dnmt3a-dependent promoter and long non-coding RNAs (lncRNAs) were methylated, which decreased the synthesis of miR-17 [125,126,127]. It has been discovered that phthalates interact with miRNAs that target mRNAs linked to processes associated with adverse outcomes in human patients, such as angiogenesis, apoptosis, and connective tissue proliferation [127,128]. Another group suggested that EDCs may exert their effects through epigenetic changes to mitochondrial DNA [129]. Some of the nongenomic AhR pathways of activation that have been linked to mitogenic responses are increased human epidermal growth factor receptor 2 (HER2/neu), v-myc myelocyto-matosis viral oncogene homologue (c-myc), FBJ murine esteosarcoma viral oncogene homologue (c-fos), jun proto-oncogene (c-jun), Harvey rat sarcoma viral onco-gene homologue (Ha-ras), cyclin-dependent kinase 4 (CDK4), and nuclear factor kappa-B (NF-kB) [68e70] [125,129]. Telomerase activity is stimulated by C-Myc expression through AhR signalling and/or ER-independent mechanisms [130,131]. Through the Plyc, Mekk, IRAK, and PLC-β signalling pathways, phthalates alter gene expression in breast cancer (Figure 7) [121,122,123,124,125,126,127,128,129]. Another nongenomic AhR-activated factor implicated in carcinogenesis is cyclooxygenase-2 (Cox-2) [123,125]. The overexpression of COX-2 and elevated levels of prostaglandin E2 (PGE2) is associated with breast carcinogenesis [122,123,124,125]. These changes can influence the expression of genes linked to breast cancer, including AHR, BAX, BCL2, CAT, ESR2, IL6, and PTGS2 [120,121,122,123,124,125,126,127,128,129,130]. Understanding the impact of phthalates in this situation is critical for establishing preventive measures and therapeutic treatments [120,121,122,123,124,125,126,127,128,129,130]. More research is needed to completely understand the processes through which these substances contribute to breast cancer risk and to identify means of reducing their effects.

### 2.6. Polychlorinated Biphenyls

**Figure 8:**
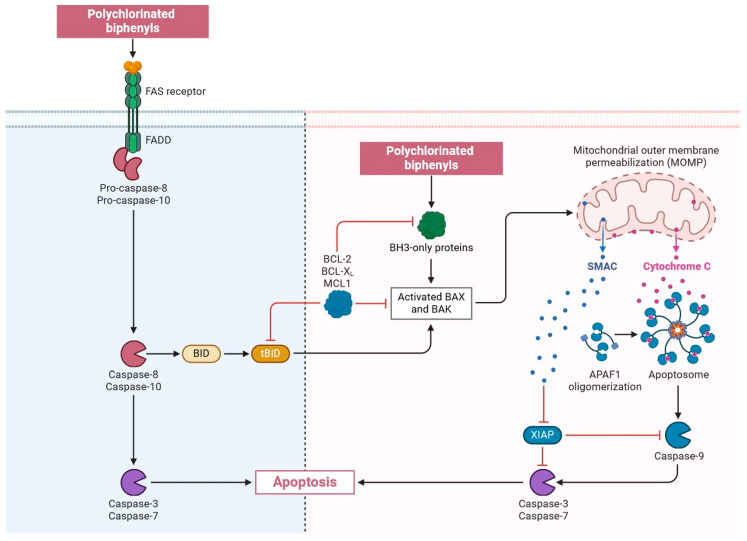
PCB activates relevant upstream signalling cascades, including p38, extracellular regulated protein kinases (ERK), and mitogen-activated protein kinase (MAPK). This includes phosphatidylinositol-4,5-bisphosphate 3-kinase (PI3K)/Akt. Reactive oxygen species (ROS) were produced following a PCB challenge and antioxidant therapy, which significantly reduced the activation of these axis signalling pathways by PCBs and caused breast cancer metastasis and progression. This interaction underscores the potential for targeted therapies that could disrupt these pathways, offering new avenues for treatment in patients affected by PCB-related breast cancer (the figure was designed using BioRender graphics: https://www.biorender.com, accessed on 14 August 2024).

It has been established that multiple receptors and enzyme networks interact with PCBs, and recent research has examined the possibility that PCBs may alter the endocrine system [131,132]. The cellular microenvironment responds to stimuli such as food availability, hypoxia, and extracellular pH and can epigenetically change cancer cells’ metabolic behavior to adapt to changing environments [132,133]. The fact that cancer cells’ metabolic profiles differ from those of normal cells reveals the underlying genetic and epigenetic machinery that is altered in breast cancer, giving cancer cells a growth advantage for survival [134,135]. Cellular metabolites moving between cellular compartments such as the cytoplasm, mitochondria, and nucleus have the potential to regulate gene expression by altering the availability of enzymatic substrates and cofactors required for metabolic reactions that mediate epigenetic processes such as DNA and histone modifications [136,137]. Metabolites that move between different parts of a cell, like the nucleus, mitochondria, and cytoplasm, can change gene expression by affecting the availability of enzyme substrates and cofactors needed for metabolic reactions that control epigenetic processes like DNA and histone modifications [137,138]. In addition, it has been demonstrated that PCBs that are not dioxin-like and those that are dioxin-like have different effects on DNA methylation; the former promote hypermethylation, while the latter stimulate hypermethylation [138,139]. The co-chaperone protein p23 and the chaperone protein hsp90 interact with the AhR-containing cytoplasmic Per-Ahreceptor Nuclear Translocator (ARNT)-Sim domain protein family [139,140]. AhR plays a major role in the development of cancer because it divides and travels into the nucleus when dL-PcBs activate the ligand [135,136,137,138]. There, it connects to dNA response elements via an ARNT to initiate transcription and AhR activation. The calcium 2+-regulated ion channel RyR is a component of both the endoplasmic reticulum (ER) in non-muscle cells and the sarcoplasmic reticulum (SR) in muscle cells [134,135,136,137,138,139,140]. RyR activation can swiftly release Ca^2+^ from the ER/SR. An NdL-PcB and a single congener increased RyR activity by 2.4–19.2 times [137,138,139,140,141,142]. There is evidence connecting NdL-PcBs to the development of cancer. Both Ca^2+^ and MAPK signalling are important in the development of breast cancer. PCBs trigger pertinent upstream signalling cascades, such as MAPK, ERK, and p38 [137,138,139,140,141,142,143,144]. ROS are generated after the PCB challenge and greatly decrease the activation of these axis signalling pathways by the PCBs, which promotes breast cancer metastasis (Figure 8) [131,132,133,134,135,136,137,138,139,140,141,142,143,144,145]. These modifications can influence chromatin structure, ultimately affecting gene accessibility and transcriptional activity. Consequently, the interplay between cellular metabolism and gene expression underscores the importance of metabolic pathways in cellular function and identity.

### 2.7. Parabens

**Figure 9:**
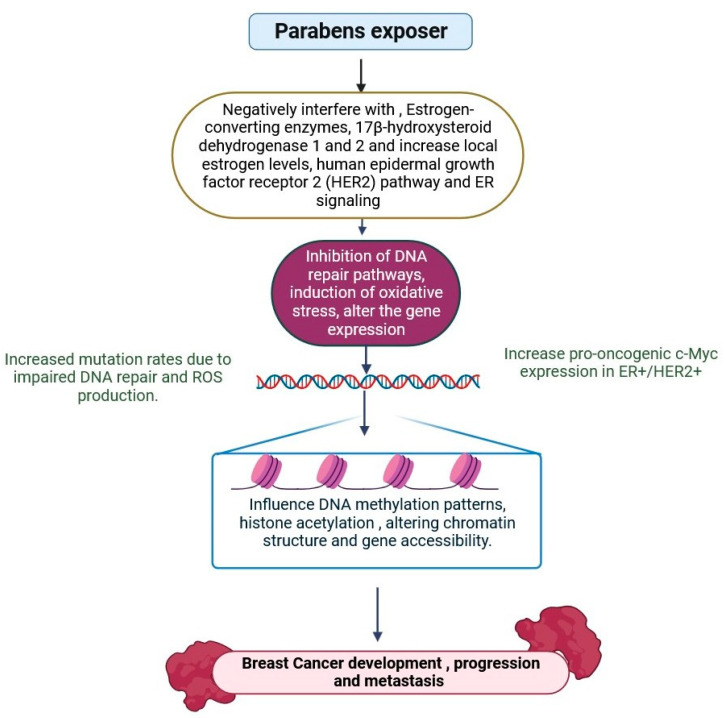
Epigenetic changes can influence the expression of genes involved in paraben metabolism, detoxification, and response. Parabens, including methyl paraben and propylparaben, are weak estrogen mimics that attach to estrogen receptors. Aberrant hypermethylation of CpG islands in promoter regions can mute genes that metabolise or detoxify paraben, including UDP-glucuronosyl transferees (UGTs) and sulfotransferases. Their estrogenic action may impact breast cancer cell proliferation, especially in ER-positive tumours. Parabens may affect the expression of microRNA (miR-155, miR-21), which regulate genes involved in cell cycle control, apoptosis, and estrogen response (the figure was designed using BioRender graphics: https://www.biorender.com, accessed on 16 August 2024).

Parabens are a type of EDC that can mimic estrogen in the body and are used as preservatives in hair and personal care products [146]. Hair and personal care products are thought to be the most common sources of paraben exposure, though other sources may exist [147]. The Environmental Working Group (EWG) created a hazardous chemical scale for cosmetic, personal care, and household product chemicals. Consumers can rate the hazard level of their items on a scale from 1 (best) to 10 (worst) [146,147]. Hormonally dangerous substances have been related to breast cancer; in particular, investigations have discovered oestrogenic qualities in paraben-containing hair and other personal care products extensively promoted to Black women [146,147,148]. Parabens are alkyl ester forms of p-hydroxybenzoic acid used in everyday products to inhibit the growth of hazardous pathogens and moulds [149]. Parabens are believed to enhance the prevalence of breast cancer (BC); nevertheless, very limited studies have examined the interactions between parabens, global DNA methylation (DNAm), and BC risk. Despite scant epidemiological data linking paraben exposure to breast cancer, recent in vitro and animal model studies have provided light on parabens’ endocrine-modulating activities, implying that parabens may be involved in breast carcinogenesis (Figure 9) [71,72,150]. The relationships between parabens and BCs have been determined according to the tumour promoter methylation state of 13 genes with documented functions in breast carcinogenesis that are often methylated in breast tumour tissues and linked to the malignant BC phenotype. These comprise the promoter regions of steroid hormone genes (ESR1, PGR, and RARβ), tumour suppressor genes (APC, BRCA1, CDH1, DAPK1, HIN1, P16, and RASSF1a), an oncogene (CCND2), a detoxification gene (GSTP1), and a transcription factor (TWIST1) [9,11,14,71,146,147,148,149,150]. Further investigation is required to understand the link between parabens and tumour-promoting methylation on the epigenetic level that enhances the risk of breast cancer.

### 2.8. Organochlorine

**Figure 10:**
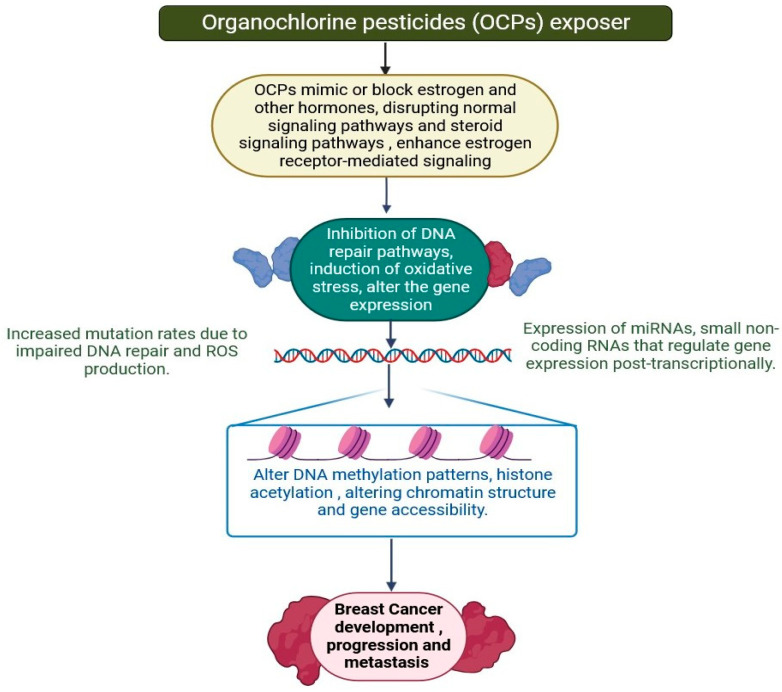
Epigenetic inactivation of organochlorine-responsive pathways in breast cancer entails suppressing cellular activity and imitating or interfering with estrogen signalling, which is crucial in hormone receptor-positive breast cancers. Aberrant methylation of gene promoters can silence detoxifying enzymes such as CYP1A1 and glutathione S-transferases (GSTs), which metabolise OCs. Epigenetically silencing genes that encode hormone receptors, such as ESR1 for ERα, can influence tumour responsiveness to estrogen (the figure was designed using BioRender graphics: https://www.biorender.com, accessed on 16 August 2024).

Organochlorine pesticides (OCPs) have been widely employed since the 1940s, providing significant agricultural benefits but also causing environmental harm. Since the 1980s, a majority of nations have banned the production of OC pesticides [151,152]. However, certain OC pesticides (e.g., dicofol) used to combat malaria are still permitted in some impoverished nations [153]. Because of their slow degradations, OCP compounds can persist in the food chain for extended periods of time and accumulate in a variety of biota, including humans [151,152,153]. Accumulating evidence suggested a link between OCP exposure and breast cancer risk [152,153,154,155]. Previous research has shown that long-term exposure to OCPs, such as in the workplace, is associated with an increased risk of multiple types of cancer, which may explain the negative effects of OCP exposure, such as endocrine disruption, oxidative stress induction, and epigenetic modifications [151,152,153,154]. OCPs can change DNA methylation patterns, which are important for controlling gene expression [155,156]. For example, tumour suppressor genes may be hypermethylated or oncogenes may be hypomethylated, increasing the risk of cancer. Studies have found altered methylation patterns in breast tissue exposed to OCPs, which may be suppressing genes essential in DNA repair and cell cycle regulation [11,72,157]. OCP exposure has been demonstrated to modify levels of gene promoter methylation. Another prominent epigenetic modification is histone modifications, which include changes to histone N-tail residues [14,20,154,155,156,157]. Some studies have shown that OCPs can change histone acetylation and methylation status, which, when combined with DNA methylation, can impact chromatin shape and, as a result, transcription activity [152,153,154,155,156,157]. In breast cancer patients, researchers have looked at the methylation patterns of two tumour suppressor genes, RRP22 and P16, as well as histone modifications such as methylation at lysine 4 of histone H3 (H3K4), acetylation at lysine 9 of histone H3 (H3K9), acetylation at lysine 16 of histone H4 (H4K16), and methylation at lysine 20 of histone H4 (H4K20) [20,72,157]. The expression of the genome and epigenome is limited to breast cancer, and the mechanisms of inactivation have yet to be completely investigated [153,154,155,156,157]. The epigenetic inactivation of organochlorine-responsive pathways in breast cancer entails lowering cellular activity while imitating or interfering with estrogen signalling, which is critical in hormone receptor-positive breast cancers. The aberrant methylation of gene promoters can inhibit detoxifying enzymes like CYP1A1 and glutathione S-transferases (GSTs), which metabolise OCPs. Silencing genes that encode hormone receptors, like ESR1 for ERα, can affect tumour response to estrogen. Furthermore, multiple studies have found changes in the methylation and acetylation of histones H3 and H4 in cancer, underlining the significance of epigenetic modifications in breast cancer growth (Figure 10) [72,151,152,153,154,155,156,157]. These changes can impact gene expression patterns that promote tumour growth and metastasis, indicating prospective therapeutic targets and the need for more research into epigenetic control in cancer treatment tactics.

### 2.9. Dioxins

**Figure 11:**
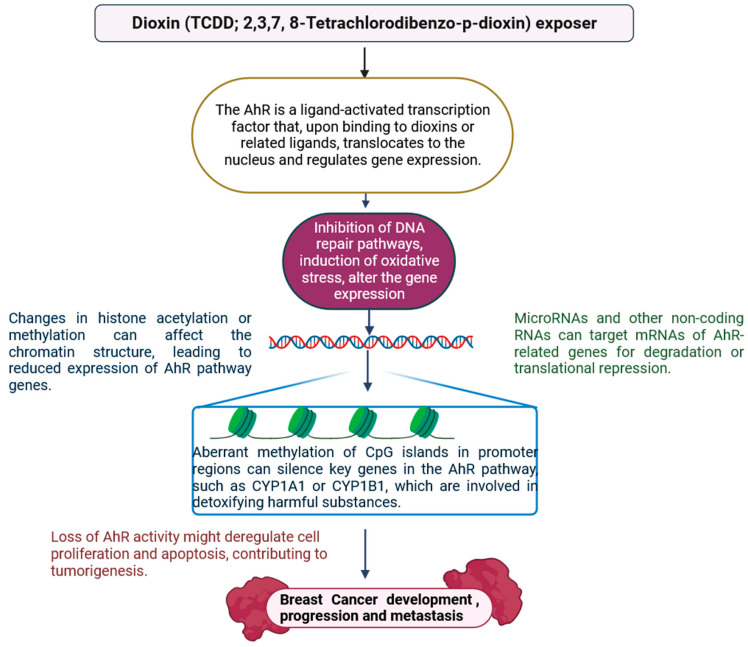
The AhR is a ligand-activated transcription factor that regulates gene expression by translocating to the nucleus after binding to dioxins or similar ligands. Aberrant methylation of CpG islands in promoter regions has the potential to silence critical AhR pathway genes such as CYP1A1 and CYP1B1, which are involved in the detoxification of hazardous drugs. Loss of AhR function may disrupt cell proliferation and apoptosis, contributing to carcinogenesis in breast cancer (the figure was designed using BioRender graphics: https://www.biorender.com, accessed on 19 August 2024).

Dioxins (TCDD: 2,3,7,8-Tetrachlorodibenzodioxin) stimulates CYP1A1 by attaching to and activating the aryl hydrocarbon receptor (AhR), which then moves to the nucleus and binds with its companion protein Arnt to form an active heteromeric transcription factor (AhRC for AhR complex) [158]. AhRC regulates TCDD-inducible gene expression by interacting with DNA-binding sites known as dioxin response elements (DRE) on the CYP1A1 enhancer [70,73,159]. TCDD stimulates CYP1A1 by attaching to and activating the aryl hydrocarbon receptor (AhR), which then moves to the nucleus and binds with its companion protein Arnt to form an active heteromeric transcription factor (AhRC for AhR complex) [5,6,7,8,74,160]. All DRE sites contain a CpG dinucleotide, which, when methylated in vitro, decreases AhRC interaction in an electrophoretic mobility shift experiment and reduces TCDD-inducible reporter gene expression [13,74,160,161,162,163,164]. Several genes in breast cancer exhibit CpG island hypermethylation, and in several instances, aberrant activity of DNA methyltransferases led to the hypermethylation and silencing of HOXA5, TMS1, p16, RASSF1A, and BRCA1 genes with tumour suppressor behavior [11,13,75,161,162,163]. Furthermore, promoter hypermethylation silences genes such as E-cadherin, TMS1, GSTP1, and p16 [21,70,74,158,164]. These genes are involved in numerous biological processes, including estrogen signalling, pro-apoptosis (HOXA5, TMS1), cell cycle checkpoints (RASSF1A, p16), and DNA repair pathways (BRCA1) [161,162,163,164]. The BRCA1 gene is one of the greatest instances of a breast cancer susceptibility gene that is commonly repressed in sporadic breast tumours; yet, the CpG hypermethylation of BRCA1 has been linked to DNMT 3b overexpression. Early stages of sporadic breast cancer show a loss of the cell cycle checkpoint gene p16INK4a due to aberrant CpG promoter methylation, and approximately 80% of breast tumours show the decreased expression of another cell cycle inhibitor gene, p21/CIP1/WAF1, due to increased methylation of the p21/CIP1/WAF1 gene (Figure 11) [74,75,158,159,160,161,162,163,164]. These results indicate that methylation of the CYP1A1 enhancer may reduce its TCDD response. These findings suggest that methylation of the CYP1A1 enhancer may diminish its TCDD response, thereby affecting cellular proliferation and tumour growth [158,159,160,161]. More research is needed to understand the mechanisms by which these epigenetic changes contribute to breast cancer development and to identify possible therapeutic targets for intervention.

## 3. Impact of EDCs on Dietary Intake in Breast Cancer

Women with low breast cancer risk are rapidly more sensitive to the illness compared to those with high-risk factors [165]. This increased sensitivity may be attributed to a variety of biological and environmental influences that affect their overall health and immune response [166]. Excessive doses of genistein have been shown in vitro to promote cell proliferation in tumours that are reliant on estrogen. Further discoveries have indicated that isoflavone-induced promoter demethylation in breast epithelial cells may target RARb2 (RARB) and CCND2 [166,167]. In breast cancer cells, genistein may reduce the expression and activity of all three DNA methyl transferase enzymes, which would deplete de novo methylation and preserve DNA methylation [168]. Additionally, methyl-binding domain protein 2’s variable binding to the BRCA1 promoter is increased by resveratrol, preventing gene suppression in MCF-7ER-positive breast cancer cells [168]. In a mouse xenograft model, resveratrol raised miR-141 and miR-200c, which decreased the number of cancer stem cells [169]. In breast cancer cell lines, resveratrol also increased the expression of many additional tumour-suppressive miRNAs, such as miR-16 and miR-143 [170]. There was a notable loss of 5 hydroxymethylation of cytosine (5hmC) and 5 methylation of cytosine (mC) in the epithelial cells of the TEBs, a dramatic loss of the histone H4 lysine 20 monomethylation (H4K20me1) mark in these cells, and ER a-positive-associated cell proliferation in the epithelial cells of the TEBs (proliferating epithelial cells are known to have low numbers of ER a-positive cells) [171]. The incidence of DMBA-induced cancer is significantly higher in correlation with these early cellular and epigenetic changes [172,173,174,175,176]. Moreover, current studies suggest that a diet heavy in butter and safflower oil elicits a response to BPA more effectively than a diet heavy in olive oil [173,174,175,176,177]. The four families of phytoestrogens—isoflavones, lignans, coumestans, and stilbens—compose more than 100 different chemicals that have been identified in various plant sources. These compounds can mimic estrogen in the body, potentially influencing hormonal balance and offering various health benefits, including reduced risks of certain cancers and the alleviation of menopausal symptoms [170,171,172,173,174,175,176,177]. Various epidemiological studies have demonstrated that consuming a diet high in soy or taking supplements lowers the risk of breast cancer. Research conducted on animals has demonstrated the diverse effects of isoflavones, such as genistein, on the growth and maturation of the mammary gland from early childhood to maturity [177]. Crucially, MiR-93 was epigenetically repressed, which led to increased cell proliferation by blocking the apoptotic pathway mediated by p53 [178]. Studies in the breast and other tissues provide a solid foundation for future investigations into global epigenetic changes in the breasts of children exposed to DES in utero, even if there is presently insufficient information to suggest an epigenetic basis for DES-induced breast cancer risk [19,179,180,181]. A well-known organochlorine, 2, 3, 7, 8-tetrachloridibenzop-dioxin (2, 3, 7, 8-TCDD), is a pollutant that is created during chemical combustion and manufacture [180,181]. TCDD is categorised as an endocrine disruptor with significant anti-estrogenic effects and a substantial affinity for the aromatic hydrocarbon receptor (AhR) [182,183]. Rats exposed to TCDD during pregnancy showed a reduction in lobules and an increase in TEBs [184]. According to research conducted at the molecular level with the Holtzman rats model, TCDD may act systemically by elevating the expression of Esr1mRNA in the ovaries, uterus, and breast while reducing the generation of oestradiol [185].Chemicals employed for printed circuit boards (PCBs) include plasticisers, solvents, hydraulic fluids, and printing inks. It has been discovered that PCBs accumulate in breast adipose tissue before entering breast milk [186]. Epidemiology studies have found a link between PAH-DNA adducts and the incidence of breast cancer. Studies have suggested that the site of preference for PAH-induced DNA adducts formation may be methylated CpG sites. Various studies have found a favourable correlation between active or passive smoking and an increased risk of breast cancer [187]. The chemical perfluorooctanoic acid (PFOA) is widely used in business and the environment for a variety of purposes, including surfactants, water proofing, insulating agents, and dental goods [188]. PFOA is a non-lipophilic protein-binding material that persists in humans for 2–4 years and in mice for 16–22 days [188]. Given that preeclampsia, early menopause, and delayed puberty have all been demonstrated to lessen the risk of breast cancer, epidemiological research has discovered that PFOA is positively connected with these conditions and has thus been linked to a lower risk of breast cancer [189]. Additional research on PFOAs in animal models revealed modifications to the development of the mammary gland, which may increase susceptibility to carcinogens [190]. Thus, the high-throughput screening of gene promoters that target the epigenome would yield the most accurate assessment of PFOA epigenetic targets [191]. Even though no studies using mouse or human breast models have been done in this area, it is still beneficial to look into this issue. DDT is a synthetic pesticide that is widely used, has a long half-life, and is lipophilic [192]. As a result, it is a major environmental contaminant that accumulates in the fat stores of both humans and animals. Human blood still contains large concentrations of 1,1-dichloro-2,2-bis(p-chlorophenyl) ethylene (DDE), a metabolite of DDT [193]. Description of endocrine-disrupting food chemical contaminants with their origin and main source of dietary exposure has shown in Table 2 [194,195,196,197,198,199,200,201,202,203]. Human disorders, including testicular tumours, type 2 diabetes, endometrial cancer, pancreatic cancer, and breast cancer, have been linked to DDT and its derivatives, including DDE [194]. Early life exposure greatly affects the epigenetic actions of EDCs in disease states like breast cancer. Additionally, recent studies have suggested that DDT may have a critical role in the control of miRNA in the breast [195,196]. As a pesticide used on fruits and vegetables to protect them from various fungi, the majority of exposure occurs through the consumption of leftover contaminated food and beverages [197]. On the other hand, not much epidemiological study has been done about the association between breast cancer and vinclozolin exposure. Therefore, more research is required on the transgenerational epigenetic targets that induce breast tumours, which have not yet been thoroughly defined.

## 4. Conclusions and Future Prospective

EDCs regulate a variety of epigenetic mechanisms, including DNA methylation, histone modifications, and non-coding RNA expression. The same EDC may have various epigenetic effects in different tissues, making it difficult to generalise findings on pathway activation unique to breast tissue. Epigenetic modifications are reversible and can fluctuate in response to environmental factors, complicating the discovery of stable controls. The function and clinical significance of EDCs in the progression of breast cancer are elucidated in this manuscript, highlighting the molecular mechanisms through which these endocrine-disrupting chemicals influence tumour growth and metastasis. By understanding these pathways, targeted therapeutic strategies may be developed to mitigate the risks associated with EDC exposure. Breast cancer research has increasingly focused on understanding the limitations of epigenetic mechanisms influenced by endocrine-disrupting chemicals (EDCs). Many studies on EDCs and epigenetics use animal models, which may not accurately represent human breast cancer development. There have been few long-term studies tracking dietary EDC exposure and epigenetic changes over time, making it difficult to establish causal links. These mechanisms may not fully account for the complex interplay of genetic, environmental, and lifestyle factors that contribute to breast cancer risk, highlighting the need for a more comprehensive approach to studying EDCs and their long-term effects on hormonal regulation and tumour development. Studies on the relationship between EDC exposure and breast cancer risk have yielded conflicting results, which could be attributed to discrepancies in study designs, sample sizes, and other variables. Future studies on environmental epigenetic need to include different parameters by using advanced animal models. However, these systems are extremely interconnected, and the precise paths that connect them to breast cancer remain unknown. To establish causal links and inform prevention and treatment efforts, interdisciplinary approaches are required, such as more sophisticated model systems, improved exposure assessment methodologies, and longitudinal human investigations. Furthermore, future studies should examine lifestyle factors, such as circadian cycles, food, and exercise, as exposure modifiers. Epigenetic reprogramming might offer significant insights into human diversity. The primary prevention of environmental illnesses is feasible since epigenetics may be reversible. Assessing these epigenetic changes over the course of a lifetime would be difficult; thus, environmental epigenetic indicators are a better choice for determining a person’s future risk of an illness like breast cancer.

## Figures and Tables

**Table 1:** Epigenetic effects regulated by EDCs in breast cancer.

S. N.	Endocrine Disruptor	Mode of Exposer	Epigenetic Effect on Breast Cancer	Epigenetic Effect on Other Tissues	Affected Signalling Mechanism	References
1	Cadmium	As a naturally occurring element of the Earth’s crust, tobacco smoke and food contain cadmium.	Modified methylation of CCT3 chaperonin containing TCP1 subunit 3 (CCT3) and Thioredoxin Reductase (TXNRD1).	Thyroid, pancreas, prostate, and kidney.	Important signalling pathways such the NF-κB, p53, and MAPK (Mitogen-activated protein kinase) pathways are impacted by Cd^2+^, either directly or indirectly.	[30,33]
2	Arsenic	Arsenic is a common component in semiconductor circuits and lead alloys that are employed in batteries and arms.	Matrix metalloproteinas-2 (MMP2) and MMP-9 (Matrix metalloproteinase) are TNFR (tumour necrosis factor receptor) family members.	The highest associations between long-term exposure to arsenic and cancer are seen in bladder, lung, and skin cancers. Liver and kidney.	Rac, Rho, and MEKK 1–4 (mitogen-activated protein kinase kinase kinase-1) mediate JNK activation, whereas Ras/Raf/Mek signalling activates ERKs and p38 signalling. The MAPK cascade is responsible for arsenic poisoning.	[30]
3	Lead	Lead (Pb) is found naturally in the Earth’s crust.	WNT signalling epigenetic modification in ER+ breast cancer, SFRP and DKK are two examples of WNT antagonist genes whose epigenetic silencing leads to breast cancer.	Blood, digestive organs, brain, nerves, and more.	Lead causes chromatin aggregation through histone-DNA cross-links and DNA structural instability.	[32,33]
4	Bisphenol A (BPA)	Dental sealants, thermal paper, epoxy resins, and plastics.	Modifications in CDNK2A (cyclin-dependent kinase inhibitor 2A), THBS1 (Thrombospondin 1), BRCA1, CCNA1, LAMP3, TNFRSF10C, and TNFRSF10D methylation.	Increased brain tissue DNMT activity.	The activation of AKT and ERK1/2 is necessary for the proliferative and prosurvival effects of BPA in breast cancer.	[34,35]
5	Phthalates	Utilised as liquid plasticisers, which are used to make wall covers, tiles for the floor, pacifiers for teethers, toys, furniture fabric and mattresses, textiles, household goods, and medical equipment.	Kcnk5, COX-2/PGE2, PPARa, HER2/neu, v-myc, and c-myc.	The male reproductive system may be harmed by phthalate.	PKA is short for cyclin-dependent kinase (CDK). AhR-HDAC6-c-Myc and COX-2/PGE2 pathways are activated.	[36,37]
6	Polychlorinated biphenyls (PCBs)	Agents for heat transfer and coolants.	The AHRR gene’s methylation	Increased DNMT and SAM content, which raises methylation in rat liver cells	PCBs cause dysregulatory thyroid hormone and impairment of intracellular calcium signalling.	[38,39]
7	Diethylstilbestrol (DES)	A nonsteroidal oestrogen drug is diethylstilbestrol, often known as stilboestrol or stilboestrol.	Elevation of H3 trimethylation through EZH2 expression increases.	Hox gene methylation pattern in mouse endometrium; higher expression of Dnmt1 in mouse uterus.	DES and E2 share a number of pathways, such as the oestrogen receptor pathway and mammary gland development.	[40,41]
8	Polycyclic aromatic hydrocarbons (PAHs)	Incomplete combustion of materials such as coal, wood, cigarettes, and petroleum oil.	Generates DNA adducts close to breast epithelial methylation sites.	Lung cancer.	The stimulation of cytochrome P450 to produce DNA. The main factor contributing to PAH-induced carcinogenesis is epigenetic changes.	[42,43]

**Table 2:** Description of endocrine-disrupting food chemical contaminants with their origin and main source of dietary exposure.

S.N.	Group of Chemicals	Agent	Origin	Main Source of Dietary Exposure	References
1	Cd^2+^	Cadmium and cadmium compounds	Erosion, weathering, river transport, volcanic activity, and human actions including burning trash, smoking cigarettes, burning metal ore, using fossil fuels, and industrial pollution	Products from agriculture, seafood, shellfish	[197]
2	As	Agricultural products, especially rice	Occurring naturally in the soil, highly released by forest fires, volcanic eruptions, rock erosion, human activities, and chemicals such as soap, paint, dye, metal, medication, semi-conductors, fertilisers, and pesticides	Agricultural goods, particularly rice	[198]
3	Pb	Methylmercury compounds	Cans of food, water pipes, polluted water, paint, cosmetics, batteries, fuel, traditional medicine, Pb-crystal, kids’ toys, vinyl lunchboxes, and cigarettes	Seafood, poultry	[199]
4	Bisphenol	Tetrabromobisphenol A	Epoxy resins and polycarbonate plastics	Canned food	[200]
5	Phthalate	Di (2-ethylhexyl) phthalate	Detergents, plasticisers, and pesticides	Cereals, veggies, and legumes	[201]
6	Biphenyls	Polychlorinated biphenyls that resemble dioxin	Development, application, and removal of items treated with PCBs, accidental emissions resulting from burning operations, and re-emission of PCBs from water, sediment, and soil in environmental reservoirs	Seafood, meat, dairy, and fats	[202]
7	Dibenzofurans and dioxins	2,3,7,8-Tetrachlorodibenzo-para-dioxin	Byproducts in the processes of production and disposal (organochlorides) Manufacturing, bleaching of paper, and burning of materials containing chloride	Eggs, seafood, dairy products, and adipose tissue from cows	[203]

## Data Availability

No new data were created or analyzed in this study.

## References

[1] https://www.who.int/news-room/fact-sheets/detail/breast-cancer.

[2] Eremici I., Borlea A., Dumitru C., Stoian D. (2024). Breast Cancer Risk Factors among Women with Solid Breast Lesions. Clin. Pract..

[3] Knower K.C., To S.Q., Leung Y.-K., Ho S.-M., Clyne C.D. (2014). Endocrine Disruption of the Epigenome: A Breast Cancer Link. Endocr.-Relat. Cancer.

[4] Eve L., Fervers B., Le Romancer M., Etienne-Selloum N. (2020). Exposure to Endocrine Disrupting Chemicals and Risk of Breast Cancer. Int. J. Mol. Sci..

[5] Masi M., Racchi M., Travelli C., Corsini E., Buoso E. (2021). Molecular Characterization of Membrane Steroid Receptors in Hormone-Sensitive Cancers. Cells.

[6] Jiang Y., Li Y. (2024). Nutrition Intervention and Microbiome Modulation in the Management of Breast Cancer. Nutrients.

[7] Alavian-Ghavanini A., Rüegg J. (2018). Understanding Epigenetic Effects of Endocrine Disrupting Chemicals: From Mechanisms to Novel Test Methods. Basic. Clin. Pharmacol. Toxicol..

[8] Pitto L., Gorini F., Bianchi F., Guzzolino E. (2020). New Insights into Mechanisms of Endocrine-Disrupting Chemicals in Thyroid Diseases: The Epigenetic Way. Int. J. Environ. Res. Public Health.

[9] Bolt M.J., Singh P., Obkirchner C.E., Powell R.T., Mancini M.G., Szafran A.T., Stossi F., Mancini M.A. (2021). Endocrine Disrupting Chemicals Differentially Alter Intranuclear Dynamics and Transcriptional Activation of Estrogen Receptor-α. iScience.

[10] Hall J.M., Greco C.W. (2020). Perturbation of Nuclear Hormone Receptors by Endocrine Disrupting Chemicals: Mechanisms and Pathological Consequences of Exposure. Cells.

[11] Sabry R., Yamate J., Favetta L., LaMarre J. (2019). MicroRNAs: Potential Targets and Agents of Endocrine Disruption in Female Reproduction. J. Toxicol. Pathol..

[12] Tsuchida T., Kubota S., Kamiuezono S., Takasugi N., Ito A., Kumagai Y., Uehara T. (2024). Epigenetic Regulation of CXC Chemokine Expression by Environmental Electrophiles Through DNA Methyltransferase Inhibition. Int. J. Mol. Sci..

[13] Derghal A., Djelloul M., Trouslard J., Mounien L. (2016). An Emerging Role of Micro-RNA in the Effect of the Endocrine Disruptors. Front. Neurosci..

[14] Shree N., Ding Z., Flaws J., Choudhury M. (2022). Role of microRNA in Endocrine Disruptor-Induced Immunomodulation of Metabolic Health. Metabolites.

[15] Alwadi D., Felty Q., Yoo C., Roy D., Deoraj A. (2023). Endocrine Disrupting Chemicals Influence Hub Genes Associated with Aggressive Prostate Cancer. Int. J. Mol. Sci..

[16] Wallis D., Truong L., La Du J., Tanguay R., Reif D. (2021). Uncovering Evidence for Endocrine-Disrupting Chemicals That Elicit Differential Susceptibility Through Gene-Environment Interactions. Toxics.

[17] Caserta D., De Marco M.P., Besharat A.R., Costanzi F. (2022). Endocrine Disruptors and Endometrial Cancer: Molecular Mechanisms of Action and Clinical Implications, a Systematic Review. Int. J. Mol. Sci..

[18] Benevolenskaya E.V., Islam A.B.M.M.K., Ahsan H., Kibriya M.G., Jasmine F., Wolff B., Al-Alem U., Wiley E., Kajdacsy-Balla A., Macias V. (2016). DNA Methylation and Hormone Receptor Status in Breast Cancer. Clin. Epigenet.

[19] Ennour-Idrissi K., Dragic D., Issa E., Michaud A., Chang S.-L., Provencher L., Durocher F., Diorio C. (2020). DNA Methylation and Breast Cancer Risk: An Epigenome-Wide Study of Normal Breast Tissue and Blood. Cancers.

[20] Lu X., Fraszczyk E., Van Der Meer T.P., Van Faassen M., Bloks V.W., Kema I.P., Van Beek A.P., Li S., Franke L., Westra H.-J. (2020). An Epigenome-Wide Association Study Identifies Multiple DNA Methylation Markers of Exposure to Endocrine Disruptors. Environ. Int..

[21] Montjean D., Neyroud A.-S., Yefimova M.G., Benkhalifa M., Cabry R., Ravel C. (2022). Impact of Endocrine Disruptors upon Non-Genetic Inheritance. Int. J. Mol. Sci..

[22] Buoso E., Masi M., Racchi M., Corsini E. (2020). Endocrine-Disrupting Chemicals’ (EDCs) Effects on Tumour Microenvironment and Cancer Progression: Emerging Contribution of RACK1. Int. J. Mol. Sci..

[23] Maddalon A., Masi M., Iulini M., Linciano P., Galbiati V., Marinovich M., Racchi M., Buoso E., Corsini E. (2022). Effects of endocrine active contaminating pesticides on RACK1 expression and immunological consequences in THP-1 cells. Environ. Toxicol. Pharmacol..

[24] Masi M., Maddalon A., Iulini M., Linciano P., Galbiati V., Marinovich M., Racchi M., Corsini E., Buoso E. (2022). Effects of endocrine disrupting chemicals on the expression of RACK1 and LPS-induced THP-1 cell activation. Toxicology.

[25] Buoso E., Kenda M., Masi M., Linciano P., Galbiati V., Racchi M., Dolenc M.S., Corsini E. (2021). Effects of Bisphenols on RACK1 Expression and Their Immunological Implications in THP-1 Cells. Front. Pharmacol..

[26] Buoso E., Masi M., Galbiati V., Maddalon A., Iulini M., Kenda M., Sollner Dolenc M., Marinovich M., Racchi M., Corsini E. (2020). Effect of estrogen-active compounds on the expression of RACK1 and immunological implications. Arch. Toxicol..

[27] Sellami A., Montes M., Lagarde N. (2021). Predicting Potential Endocrine Disrupting Chemicals Binding to Estrogen Receptor α (ERα) Using a Pipeline Combining Structure-Based and Ligand-Based in Silico Methods. Int. J. Mol. Sci..

[28] Domingo-Relloso A., Riffo-Campos A.L., Haack K., Rentero-Garrido P., Ladd-Acosta C., Fallin D.M., Tang W.Y., Herreros-Martinez M., Gonzalez J.R., Bozack A.K. (2020). Cadmium, Smoking, and Human Blood DNA Methylation Profiles in Adults from the Strong Heart Study. Environ. Health Perspect..

[29] Liu D., Shi Q., Liu C., Sun Q., Zeng X. (2023). Effects of Endocrine-Disrupting Heavy Metals on Human Health. Toxics.

[30] Cirovic A., Satarug S. (2024). Toxicity Tolerance in the Carcinogenesis of Environmental Cadmium. Int. J. Mol. Sci..

[31] Islam R., Zhao L., Wang Y., Lu-Yao G., Liu L.-Z. (2022). Epigenetic Dysregulations in Arsenic-Induced Carcinogenesis. Cancers.

[32] Mani M., Kabekkodu S., Joshi M., Dsouza H. (2019). Ecogenetics of Lead Toxicity and Its Influence on Risk Assessment. Hum. Exp. Toxicol..

[33] Massányi P., Massányi M., Madeddu R., Stawarz R., Lukáč N. (2020). Effects of Cadmium, Lead, and Mercury on the Structure and Function of Reproductive Organs. Toxics.

[34] Xu F., Wang X., Wu N., He S., Yi W., Xiang S., Zhang P., Xie X., Ying C. (2017). Bisphenol A Induces Proliferative Effects on Both Breast Cancer Cells and Vascular Endothelial Cells Through a Shared GPER-Dependent Pathway in Hypoxia. Environ. Pollut..

[35] Cortes-Ramirez S.A., Ho S.-M., Leung Y.-K. (2024). Endocrine-Disrupting Chemicals: A Looming Threat to Current and Future Generations. Int. J. Mol. Sci..

[36] Hsieh T., Tsai C., Hsu C., Kuo P., Lee J., Chai C., Wang S., Tsai E. (2012). Phthalates Induce Proliferation and Invasiveness of Estrogen Receptor-Negative Breast Cancer Through the AhR/HDAC6/c-Myc Signaling Pathway. FASEB J..

[37] Fiocchetti M., Bastari G., Cipolletti M., Leone S., Acconcia F., Marino M. (2021). The Peculiar Estrogenicity of Diethyl Phthalate: Modulation of Estrogen Receptor α Activities in the Proliferation of Breast Cancer Cells. Toxics.

[38] Thakur C., Qiu Y., Fu Y., Bi Z., Zhang W., Ji H., Chen F. (2022). Epigenetics and Environment in Breast Cancer: New Paradigms for Anti-Cancer Therapies. Front. Oncol..

[39] Leti Maggio E., Zucca C., Grande M., Carrano R., Infante A., Bei R., Lucarini V., De Maio F., Focaccetti C., Palumbo C. (2024). Polyphenols Regulate the Activity of Endocrine-Disrupting Chemicals, Having Both Positive and Negative Effects. J. Xenobiot..

[40] Hilakivi-Clarke L. (2014). Maternal Exposure to Diethylstilbestrol during Pregnancy and Increased Breast Cancer Risk in Daughters. Breast Cancer Res..

[41] Zamora-León P. (2021). Are the Effects of DES Over? A Tragic Lesson from the Past. Int. J. Environ. Res. Public Health.

[42] White A.J., Chen J., Teitelbaum S.L., McCullough L.E., Xu X., Hee Cho Y., Conway K., Beyea J., Stellman S.D., Steck S.E. (2016). Sources of Polycyclic Aromatic Hydrocarbons Are Associated with Gene-Specific Promoter Methylation in Women with Breast Cancer. Environ. Res..

[43] Anditi B.C., Poór V., Szerencsés D., Szabó I., Wahr M., Kőnig-Péter A., Dergez T. (2024). Determination of PAH Contamination in Breast Milk Samples from Hungarian Volunteering Mothers, Using HPLC–FLD. Molecules.

[44] Strumylaitė L., Boguševičius A., Ryselis S., Pranys D., Poškienė L., Kregždytė R., Abdrachmanovas O., Asadauskaitė R. (2008). Association between cadmium and breast cancer. Medicina.

[45] Strumylaite L., Kregzdyte R., Bogusevicius A., Poskiene L., Baranauskiene D., Pranys D. (2019). Cadmium Exposure and Risk of Breast Cancer by Histological and Tumor Receptor Subtype in White Caucasian Women: A Hospital-Based Case-Control Study. Int. J. Mol. Sci..

[46] Tarhonska K., Lesicka M., Janasik B., Roszak J., Reszka E., Braun M., Kołacińska-Wow A., Jabłońska E. (2022). Cadmium and Breast Cancer—Current State and Research Gaps in the Underlying Mechanisms. Toxicol. Lett..

[47] Gachowska M., Dąbrowska A., Wilczyński B., Kuźnicki J., Sauer N., Szlasa W., Kobierzycki C., Łapińska Z., Kulbacka J. (2024). The Influence of Environmental Exposure to Xenoestrogens on the Risk of Cancer Development. Int. J. Mol. Sci..

[48] Brama M., Gnessi L., Basciani S., Cerulli N., Politi L., Spera G., Mariani S., Cherubini S., d’Abusco A.S., Scandurra R. (2007). Cadmium Induces Mitogenic Signaling in Breast Cancer Cell by an ERα-Dependent Mechanism. Mol. Cell. Endocrinol..

[49] Wojnarowski K., Cholewińska P., Palić D., Bednarska M., Jarosz M., Wiśniewska I. (2022). Estrogen Receptors Mediated Negative Effects of Estrogens and Xenoestrogens in Teleost Fishes—Review. Int. J. Mol. Sci..

[50] Aquino N.B., Sevigny M.B., Sabangan J., Louie M.C. (2012). The Role of Cadmium and Nickel in Estrogen Receptor Signaling and Breast Cancer: Metalloestrogens or Not?. J. Environ. Sci. Health Part C.

[51] Asara Y., Marchal J.A., Carrasco E., Boulaiz H., Solinas G., Bandiera P., Garcia M.A., Farace C., Montella A., Madeddu R. (2013). Cadmium Modifies the Cell Cycle and Apoptotic Profiles of Human Breast Cancer Cells Treated with 5-Fluorouracil. Int. J. Mol. Sci..

[52] Peana M., Pelucelli A., Chasapis C.T., Perlepes S.P., Bekiari V., Medici S., Zoroddu M.A. (2023). Biological Effects of Human Exposure to Environmental Cadmium. Biomolecules.

[53] Lordan R., Zabetakis I. (2022). Cadmium: A Focus on the Brown Crab (*Cancer pagurus*) Industry and Potential Human Health Risks. Toxics.

[54] Luparello C. (2021). Cadmium-Associated Molecular Signatures in Cancer Cell Models. Cancers.

[55] Desaulniers D., Vasseur P., Jacobs A., Aguila M.C., Ertych N., Jacobs M.N. (2021). Integration of Epigenetic Mechanisms into Non-Genotoxic Carcinogenicity Hazard Assessment: Focus on DNA Methylation and Histone Modifications. Int. J. Mol. Sci..

[56] Thévenod F. (2009). Cadmium and Cellular Signaling Cascades: To Be or Not to Be?. Toxicol. Appl. Pharmacol..

[57] Wang L.-H., Chen L.-R., Chen K.-H. (2021). In Vitro and Vivo Identification, Metabolism and Action of Xenoestrogens: An Overview. Int. J. Mol. Sci..

[58] Zhang Y., Li Y., Zhang J., Qi X., Cui Y., Yin K., Lin H. (2021). Cadmium Induced Inflammation and Apoptosis of Porcine Epididymis via Activating RAF1/MEK/ERK and NF-κB Pathways. Toxicol. Appl. Pharmacol..

[59] Schuler L.A., Murdoch F.E. (2021). Endogenous and Therapeutic Estrogens: Maestro Conductors of the Microenvironment of ER+ Breast Cancers. Cancers.

[60] Valbonesi P., Ricci L., Franzellitti S., Biondi C., Fabbri E. (2008). Effects of Cadmium on MAPK Signalling Pathways and HSP70 Expression in a Human Trophoblast Cell Line. Placenta.

[61] Almatroudi A., Allemailem K.S., Alwanian W.M., Alharbi B.F., Alrumaihi F., Khan A.A., Almatroodi S.A., Rahmani A.H. (2023). Effects and Mechanisms of Kaempferol in the Management of Cancers Through Modulation of Inflammation and Signal Transduction Pathways. Int. J. Mol. Sci..

[62] Pakjoo M., Ahmadi S.E., Zahedi M., Jaafari N., Khademi R., Amini A., Safa M. (2024). Interplay Between Proteasome Inhibitors and NF-κB Pathway in Leukemia and Lymphoma: A Comprehensive Review on Challenges Ahead of Proteasome Inhibitors. Cell Commun. Signal.

[63] Branca J.J.V., Fiorillo C., Carrino D., Paternostro F., Taddei N., Gulisano M., Pacini A., Becatti M. (2020). Cadmium-Induced Oxidative Stress: Focus on the Central Nervous System. Antioxidants.

[64] Schmidt N., Kowald L., Van Wijk S.J.L., Fulda S. (2019). Differential Involvement of TAK1, RIPK1 and NF-κB Signaling in Smac Mimetic-Induced Cell Death in Breast Cancer Cells. Biol. Chem..

[65] Qu F., Zheng W. (2024). Cadmium Exposure: Mechanisms and Pathways of Toxicity and Implications for Human Health. Toxics.

[66] Naldi A., Larive R.M., Czerwinska U., Urbach S., Montcourrier P., Roy C., Solassol J., Freiss G., Coopman P.J., Radulescu O. (2017). Reconstruction and Signal Propagation Analysis of the Syk Signaling Network in Breast Cancer Cells. PLoS Comput. Biol..

[67] Oke S.L., Hardy D.B. (2021). The Role of Cellular Stress in Intrauterine Growth Restriction and Postnatal Dysmetabolism. Int. J. Mol. Sci..

[68] Saintilnord W.N., Fondufe-Mittendorf Y. (2021). Arsenic-Induced Epigenetic Changes in Cancer Development. Semin. Cancer Biol..

[69] Virtuoso S., Raggi C., Maugliani A., Baldi F., Gentili D., Narciso L. (2024). Toxicological Effects of Naturally Occurring Endocrine Disruptors on Various Human Health Targets: A Rapid Review. Toxics.

[70] Sage A.P., Minatel B.C., Ng K.W., Stewart G.L., Dummer T.J.B., Lam W.L., Martinez V.D. (2017). Oncogenomic Disruptions in Arsenic-Induced Carcinogenesis. Oncotarget.

[71] Yu M., Zhang Y., Fang M., Jehan S., Zhou W. (2022). Current Advances of Nanomedicines Delivering Arsenic Trioxide for Enhanced Tumor Therapy. Pharmaceutics.

[72] Tam M., Zhang C., Kibriya M.G., Jasmine F., Roy S., Gao J., Sabarinathan M., Shinkle J., Delgado D., Ahmed A. (2018). A study of telomere length, arsenic exposure, and arsenic toxicity in a Bangladeshi cohort. Environ. Res..

[73] Dratwa-Kuzmin M., Hadra B.A., Oguz F., Ogret Y., Constantinescu I., Apostol D., Talangescu A., Constantinescu A.-E., Maruntelu I., Kościńska K. (2024). Telomere Length, HLA, and Longevity—Results from a Multicenter Study. Int. J. Mol. Sci..

[74] Tam L.M., Price N.E., Wang Y. (2020). Molecular Mechanisms of Arsenic-Induced Disruption of DNA Repair. Chem. Res. Toxicol..

[75] Pasha Q., Rain M., Tasnim S., Kanipakam H., Thinlas T., Mohammad G. (2023). The Telomere-Telomerase System Is Detrimental to Health at High-Altitude. Int. J. Environ. Res. Public Health.

[76] Lin L.-T., Liu S.-Y., Leu J.-D., Chang C.-Y., Chiou S.-H., Lee T.-C., Lee Y.-J. (2018). Arsenic Trioxide-Mediated Suppression of miR-182-5p Is Associated with Potent Anti-Oxidant Effects Through Up-Regulation of *SESN2*. Oncotarget.

[77] Gujrati H., Ha S., Wang B.-D. (2023). Deregulated microRNAs Involved in Prostate Cancer Aggressiveness and Treatment Resistance Mechanisms. Cancers.

[78] Medda N., De S.K., Maiti S. (2021). Different Mechanisms of Arsenic Related Signaling in Cellular Proliferation, Apoptosis and Neo-Plastic Transformation. Ecotoxicol. Environ. Saf..

[79] De Francisco P., Martín-González A., Rodriguez-Martín D., Díaz S. (2021). Interactions with Arsenic: Mechanisms of Toxicity and Cellular Resistance in Eukaryotic Microorganisms. Int. J. Environ. Res. Public Health.

[80] Wee P., Wang Z. (2017). Epidermal Growth Factor Receptor Cell Proliferation Signaling Pathways. Cancers.

[81] Hu Y., Li J., Lou B., Wu R., Wang G., Lu C., Wang H., Pi J., Xu Y. (2020). The Role of Reactive Oxygen Species in Arsenic Toxicity. Biomolecules.

[82] Qie S., Diehl J.A. (2016). Cyclin D1, Cancer Progression, and Opportunities in Cancer Treatment. J. Mol. Med..

[83] Zargari F., Rahaman M.S., KazemPour R., Hajirostamlou M. (2022). Arsenic, Oxidative Stress and Reproductive System. J. Xenobiot..

[84] Yin L., Yu X. (2018). Arsenic-Induced Apoptosis in the P53-Proficient and P53-Deficient Cells Through Differential Modulation of NFkB Pathway. Food Chem. Toxicol..

[85] Genchi G., Lauria G., Catalano A., Carocci A., Sinicropi M.S. (2022). Arsenic: A Review on a Great Health Issue Worldwide. Appl. Sci..

[86] Peng F., Liao M., Qin R., Zhu S., Peng C., Fu L., Chen Y., Han B. (2022). Regulated Cell Death (RCD) in Cancer: Key Pathways and Targeted Therapies. Signal Transduct. Target. Ther..

[87] Graham-Evans B., Tchounwou P.B., Cohly H.H.P. (2003). Cytotoxicity and Proliferation Studies with Arsenic in Established Human Cell Lines: Keratinocytes, Melanocytes, Dendritic Cells, Dermal Fibroblasts, Microvascular Endothelial Cells, Monocytes and T-Cells. Int. J. Mol. Sci..

[88] Atsaves V., Leventaki V., Rassidakis G.Z., Claret F.X. (2019). AP-1 Transcription Factors as Regulators of Immune Responses in Cancer. Cancers.

[89] Pittayapruek P., Meephansan J., Prapapan O., Komine M., Ohtsuki M. (2016). Role of Matrix Metalloproteinases in Photoaging and Photocarcinogenesis. Int. J. Mol. Sci..

[90] Rousseau M.-C., Parent M.-E., Nadon L., Latreille B., Siemiatycki J. (2007). Occupational Exposure to Lead Compounds and Risk of Cancer among Men: A Population-Based Case-Control Study. Am. J. Epidemiol..

[91] Mielke H.W., Gonzales C.R., Powell E.T., Egendorf S.P. (2022). Lead in Air, Soil, and Blood: Pb Poisoning in a Changing World. Int. J. Environ. Res. Public Health.

[92] Sanders T., Liu Y., Buchner V., Tchounwou P.B. (2009). Neurotoxic Effects and Biomarkers of Lead Exposure: A Review. Rev. Environ. Health.

[93] Olufemi A.C., Mji A., Mukhola M.S. (2022). Potential Health Risks of Lead Exposure from Early Life Through Later Life: Implications for Public Health Education. Int. J. Environ. Res. Public Health.

[94] Liao L.M., Friesen M.C., Xiang Y.-B., Cai H., Koh D.-H., Ji B.-T., Yang G., Li H.-L., Locke S.J., Rothman N. (2016). Occupational Lead Exposure and Associations with Selected Cancers: The Shanghai Men’s and Women’s Health Study Cohorts. Environ. Health Perspect..

[95] Kobets T., Smith B.P.C., Williams G.M. (2022). Food-Borne Chemical Carcinogens and the Evidence for Human Cancer Risk. Foods.

[96] Tilghman S.L., Bratton M.R., Segar H.C., Martin E.C., Rhodes L.V., Li M., McLachlan J.A., Wiese T.E., Nephew K.P., Burow M.E. (2012). Endocrine Disruptor Regulation of MicroRNA Expression in Breast Carcinoma Cells. PLoS ONE.

[97] Humphries B., Wang Z., Yang C. (2019). MicroRNA Regulation of Epigenetic Modifiers in Breast Cancer. Cancers.

[98] Stiefel C., Stintzing F. (2023). Endocrine-Active and Endocrine-Disrupting Compounds in Food—Occurrence, Formation and Relevance. NFS J..

[99] Soldado-Gordillo A., Álvarez-Mercado A.I. (2024). Epigenetics, Microbiota, and Breast Cancer: A Systematic Review. Life.

[100] Scinicariello F., Murray H.E., Moffett D.B., Abadin H.G., Sexton M.J., Fowler B.A. (2007). Lead and δ-Aminolevulinic Acid Dehydratase Polymorphism: Where Does It Lead? A Meta-Analysis. Environ. Health Perspect..

[101] Huang C.-C., Yang C.-C., Liu T.-Y., Dai C.-Y., Wang C.-L., Chuang H.-Y. (2020). Use of Generalized Additive Model to Detect the Threshold of δ-Aminolevulinic Acid Dehydratase Activity Reduced by Lead Exposure. Int. J. Environ. Res. Public Health.

[102] Georgiou-Siafis S.K., Tsiftsoglou A.S. (2023). The Key Role of GSH in Keeping the Redox Balance in Mammalian Cells: Mechanisms and Significance of GSH in Detoxification via Formation of Conjugates. Antioxidants.

[103] Obukhova L., Kopytova T., Murach E., Shchelchkova N., Kontorshchikova C., Medyanik I., Orlinskaya N., Grishin A., Kontorshchikov M., Badanina D. (2022). Relationship Between Glutathione-Dependent Enzymes and the Immunohistochemical Profile of Glial Neoplasms. Biomedicines.

[104] Steenland K., Boffetta P. (2000). Lead and Cancer in Humans: Where Are We Now?. Am. J. Ind. Med..

[105] Baierle M., Charão M.F., Göethel G., Barth A., Fracasso R., Bubols G., Sauer E., Campanharo S.C., Rocha R.C.C., Saint’Pierre T.D. (2014). Are Delta-Aminolevulinate Dehydratase Inhibition and Metal Concentrations Additional Factors for the Age-Related Cognitive Decline?. Int. J. Environ. Res. Public Health.

[106] Cariati F., Carbone L., Conforti A., Bagnulo F., Peluso S.R., Carotenuto C., Buonfantino C., Alviggi E., Alviggi C., Strina I. (2020). Bisphenol A-Induced Epigenetic Changes and Its Effects on the Male Reproductive System. Front. Endocrinol..

[107] Kwon Y. (2022). Potential Pro-Tumorigenic Effect of Bisphenol A in Breast Cancer via Altering the Tumor Microenvironment. Cancers.

[108] Salian-Mehta S., Doshi T., Vanage G. (2014). Exposure of Neonatal Rats to the Endocrine Disrupter Bisphenol A Affects Ontogenic Expression Pattern of Testicular Steroid Receptors and Their Coregulators. J. Appl. Toxicol..

[109] Nair V.A., Valo S., Peltomäki P., Bajbouj K., Abdel-Rahman W.M. (2020). Oncogenic Potential of Bisphenol A and Common Environmental Contaminants in Human Mammary Epithelial Cells. Int. J. Mol. Sci..

[110] Sugiyama K., Kinoshita M., Grúz P., Kasamatsu T., Honma M. (2022). Bisphenol-A Reduces DNA Methylation After Metabolic Activation. Genes. Environ..

[111] Besaratinia A. (2023). The State of Research and Weight of Evidence on the Epigenetic Effects of Bisphenol A. Int. J. Mol. Sci..

[112] Lan X., Fu L.-J., Zhang J., Liu X.-Q., Zhang H.-J., Zhang X., Ma M.-F., Chen X.-M., He J.-L., Li L.-B. (2017). Bisphenol A Exposure Promotes HTR-8/SVneo Cell Migration and Impairs Mouse Placentation Involving Upregulation of Integrin-Β1 and MMP-9 and Stimulation of MAPK and PI3K Signaling Pathways. Oncotarget.

[113] Wang K., Huang D., Zhou P., Su X., Yang R., Shao C., Ma A., Wu J. (2022). Individual and Combined Effect of Bisphenol A and Bisphenol AF on Prostate Cell Proliferation Through NF-κ. Int. J. Mol. Sci..

[114] Zhang X., Liu N., Weng S., Wang H. (2016). Bisphenol A Increases the Migration and Invasion of Triple-Negative Breast Cancer Cells via Oestrogen-related Receptor Gamma. Basic. Clin. Pharmacol. Toxicol..

[115] Yu M., Tang Q., Lei B., Yang Y., Xu L. (2022). Bisphenol AF Promoted the Growth of Uterus and Activated Estrogen Signaling Related Targets in Various Tissues of Nude Mice with SK-BR-3 Xenograft Tumor. Int. J. Environ. Res. Public Health.

[116] Deb P., Bhan A., Hussain I., Ansari K.I., Bobzean S.A., Pandita T.K., Perrotti L.I., Mandal S.S. (2016). Endocrine Disrupting Chemical, Bisphenol-A, Induces Breast Cancer Associated Gene HOXB9 Expression in Vitro and in Vivo. Gene.

[117] Yuan J., Yang J., Xu X., Wang Z., Jiang Z., Ye Z., Ren Y., Wang Q., Wang T. (2023). Bisphenol A (BPA) Directly Activates the G Protein-Coupled Estrogen Receptor 1 and Triggers the Metabolic Disruption in the Gonadal Tissue of *Apostichopus japonicus*. Biology.

[118] Gao H., Yang B.-J., Li N., Feng L.-M., Shi X.-Y., Zhao W.-H., Liu S.-J. (2015). Bisphenol A and Hormone-Associated Cancers: Current Progress and Perspectives. Medicine.

[119] Tiffon C. (2018). The Impact of Nutrition and Environmental Epigenetics on Human Health and Disease. Int. J. Mol. Sci..

[120] Chen F.-P., Chien M.-H. (2014). Lower Concentrations of Phthalates Induce Proliferation in Human Breast Cancer Cells. Climacteric.

[121] Liu G., Cai W., Liu H., Jiang H., Bi Y., Wang H. (2021). The Association of Bisphenol A and Phthalates with Risk of Breast Cancer: A Meta-Analysis. Int. J. Environ. Res. Public Health.

[122] Singh S., Li S.S.-L. (2012). Epigenetic Effects of Environmental Chemicals Bisphenol A and Phthalates. Int. J. Mol. Sci..

[123] Huang W., Li H., Yu Q., Xiao W., Wang D.O. (2022). LncRNA-Mediated DNA Methylation: An Emerging Mechanism in Cancer and Beyond. J. Exp. Clin. Cancer Res..

[124] Stathori G., Hatziagapiou K., Mastorakos G., Vlahos N.F., Charmandari E., Valsamakis G. (2024). Endocrine-Disrupting Chemicals, Hypothalamic Inflammation and Reproductive Outcomes: A Review of the Literature. Int. J. Mol. Sci..

[125] Abolhasanzadeh N., Sarabandi S., Dehghan B., Karamad V., Avci C.B., Shademan B., Nourazarian A. (2024). Exploring the Intricate Relationship Between miRNA Dysregulation and Breast Cancer Development: Insights into the Impact of Environmental Chemicals. Front. Immunol..

[126] Sibuh B.Z., Quazi S., Panday H., Parashar R., Jha N.K., Mathur R., Jha S.K., Taneja P., Jha A.K. (2023). The Emerging Role of Epigenetics in Metabolism and Endocrinology. Biology.

[127] Dutta S., Haggerty D.K., Rappolee D.A., Ruden D.M. (2020). Phthalate Exposure and Long-Term Epigenomic Consequences: A Review. Front. Genet..

[128] Varghese E., Liskova A., Kubatka P., Samuel S.M., Büsselberg D. (2020). Anti-Angiogenic Effects of Phytochemicals on miRNA Regulating Breast Cancer Progression. Biomolecules.

[129] Wang Y., Chen H., Long C., Tsai C., Hsieh T., Hsu C., Tsai E. (2012). Possible Mechanism of Phthalates-induced Tumorigenesis. Kaohsiung J. Med. Sci..

[130] Saftić Martinović L., Mladenić T., Lovrić D., Ostojić S., Dević Pavlić S. (2024). Decoding the Epigenetics of Infertility: Mechanisms, Environmental Influences, and Therapeutic Strategies. Epigenomes.

[131] Zimeri A.M., Robb S.W., Hassan S.M., Hire R.R., Davis M.B. (2015). Assessing Heavy Metal and PCB Exposure from Tap Water by Measuring Levels in Plasma from Sporadic Breast Cancer Patients, a Pilot Study. Int. J. Environ. Res. Public Health.

[132] da Mota T.H.A., Camargo R., Biojone E.R., Guimarães A.F.R., Pittella-Silva F., de Oliveira D.M. (2023). The Relevance of Telomerase and Telomere-Associated Proteins in B-Acute Lymphoblastic Leukemia. Genes.

[133] Pittman G.S., Wang X., Campbell M.R., Coulter S.J., Olson J.R., Pavuk M., Birnbaum L.S., Bell D.A. (2020). Polychlorinated Biphenyl Exposure and DNA Methylation in the Anniston Community Health Survey. Epigenetics.

[134] Łukasiewicz S., Czeczelewski M., Forma A., Baj J., Sitarz R., Stanisławek A. (2021). Breast Cancer—Epidemiology, Risk Factors, Classification, Prognostic Markers, and Current Treatment Strategies—An Updated Review. Cancers.

[135] Cox M.B., Miller C.A. (2004). Cooperation of Heat Shock Protein 90 and P23 in Aryl Hydrocarbon Receptor Signaling. Cell Stress. Chaperones.

[136] Montano L., Pironti C., Pinto G., Ricciardi M., Buono A., Brogna C., Venier M., Piscopo M., Amoresano A., Motta O. (2022). Polychlorinated Biphenyls (PCBs) in the Environment: Occupational and Exposure Events, Effects on Human Health and Fertility. Toxics.

[137] Brown T.M., Ross P.S., Reimer K.J., Veldhoen N., Dangerfield N.J., Fisk A.T., Helbing C.C. (2014). PCB Related Effects Thresholds as Derived Through Gene Transcript Profiles in Locally Contaminated Ringed Seals (*Pusa hispida*). Environ. Sci. Technol..

[138] Bassal M.A. (2023). The Interplay Between Dysregulated Metabolism and Epigenetics in Cancer. Biomolecules.

[139] Krasnyi A.M., Sadekova A.A., Kometova V.V., Rodionov V.V., Yarotskaya E.L., Sukhikh G.T. (2023). Methylation Profile of Small Breast Cancer Tumors Evaluated by Modified MS–HRM. Int. J. Mol. Sci..

[140] Panesar H.K., Kennedy C.L., Keil Stietz K.P., Lein P.J. (2020). Polychlorinated Biphenyls (PCBs): Risk Factors for Autism Spectrum Disorder?. Toxics.

[141] Salimi F., Asadikaram G., Ashrafi M.R., Zeynali Nejad H., Abolhassani M., Abbasi-Jorjandi M., Sanjari M. (2023). Organochlorine pesticides and epigenetic alterations in thyroid tumors. Front. Endocrinol..

[142] Lincho J., Martins R.C., Gomes J. (2021). Paraben Compounds—Part I: An Overview of Their Characteristics, Detection, and Impacts. Appl. Sci..

[143] Khan Z., Zheng Y., Jones T.L., Delaney A.A., Correa L.F., Shenoy C.C., Khazaie K., Daftary G.S. (2018). Epigenetic Therapy: Novel Translational Implications for Arrest of Environmental Dioxin-Induced Disease in Females. Endocrinology.

[144] Zhao W., Lu J., Lai Y., Hou Y., Zhao X., Wei Q., Zou X., Gou Z. (2023). Occurrences, Possible Sources, and Risk Impacts of Organochlorine Pesticides in Soil of Changchun Central Urban Area, Northeast China. Sustainability.

[145] Li Y., Tollefsbol T.O. (2010). Impact on DNA Methylation in Cancer Prevention and Therapy by Bioactive Dietary Components. CMC.

[146] Parada H., Sahrai L., Wolff M.S., Santella R.M., Chen J., Neugut A.I., Teitelbaum S.L. (2022). Urinary parabens and breast cancer risk: Modification by LINE-1 and LUMA global DNA methylation, and associations with breast cancer defined by tumor promoter methylation status. Mol. Carcinog..

[147] Azeredo D.B.C., de Sousa Anselmo D., Soares P., Graceli J.B., Magliano D.C., Miranda-Alves L. (2023). Environmental Endocrinology: Parabens Hazardous Effects on Hypothalamic–Pituitary–Thyroid Axis. Int. J. Mol. Sci..

[148] Hager E., Chen J., Zhao L. (2022). Minireview: Parabens Exposure and Breast Cancer. Int. J. Environ. Res. Public Health.

[149] Parla A., Zormpa E., Paloumpis N., Kabir A., Furton K.G., Roje Ž., Samanidou V., Vinković Vrček I., Panderi I. (2021). Determination of Intact Parabens in the Human Plasma of Cancer and Non-Cancer Patients Using a Validated Fabric Phase Sorptive Extraction Reversed-Phase Liquid Chromatography Method with UV Detection. Molecules.

[150] Takenaka Y., Watanabe M., Osaka Twin Research Group (2024). Environmental Factor Index (EFI): A Novel Approach to Measure the Strength of Environmental Influence on DNA Methylation in Identical Twins. Epigenomes.

[151] Ajiboye T.O., Kuvarega A.T., Onwudiwe D.C. (2020). Recent Strategies for Environmental Remediation of Organochlorine Pesticides. Appl. Sci..

[152] Yao S., Huang J., Zhou H., Cao C., Ai T., Xing H., Sun J. (2022). Levels, Distribution and Health Risk Assessment of Organochlorine Pesticides in Agricultural Soils from the Pearl River Delta of China. Int. J. Environ. Res. Public Health.

[153] López-Benítez A., Guevara-Lara A., Domínguez-Crespo M.A., Andraca-Adame J.A., Torres-Huerta A.M. (2024). Concentrations of Organochlorine, Organophosphorus, and Pyrethroid Pesticides in Rivers Worldwide (2014–2024): A Review. Sustainability.

[154] Bachelet D., Verner M.-A., Neri M., Cordina Duverger É., Charlier C., Arveux P., Haddad S., Guénel P. (2019). Breast Cancer and Exposure to Organochlorines in the CECILE Study: Associations with Plasma Levels Measured at the Time of Diagnosis and Estimated during Adolescence. Int. J. Environ. Res. Public Health.

[155] Lee E., Kinninger A., Ursin G., Tseng C., Hurley S., Wang M., Wang Y., Park J.-S., Petreas M., Deapen D. (2020). Serum Levels of Commonly Detected Persistent Organic Pollutants and Per- and Polyfluoroalkyl Substances (PFASs) and Mammographic Density in Postmenopausal Women. Int. J. Environ. Res. Public Health.

[156] Menouni A., Duca R.C., Berni I., Khouchoua M., Ghosh M., El Ghazi B., Zouine N., Lhilali I., Akroute D., Pauwels S. (2021). The Parental Pesticide and Offspring’s Epigenome Study: Towards an Integrated Use of Human Biomonitoring of Exposure and Effect Biomarkers. Toxics.

[157] Hoaib S., Ansari M.A., Ghazwani M., Hani U., Jamous Y.F., Alali Z., Wahab S., Ahmad W., Weir S.A., Alomary M.N. (2023). Prospective Epigenetic Actions of Organo-Sulfur Compounds against Cancer: Perspectives and Molecular Mechanisms. Cancers.

[158] Furue M., Ishii Y., Tsukimori K., Tsuji G. (2021). Aryl Hydrocarbon Receptor and Dioxin-Related Health Hazards—Lessons from Yusho. Int. J. Mol. Sci..

[159] Choi H., Ha K., Kim J.T., Moon M.K., Joung H., Lee H.K., Pak Y.K. (2024). Relationships among Dioxin-like Mitochondria Inhibitor Substances (MIS)-Mediated Mitochondria Dysfunction, Obesity, and Lung Function in a Korean Cohort. Toxics.

[160] Tue N.M., Kimura E., Maekawa F., Goto A., Uramaru N., Kunisue T., Suzuki G. (2024). Uptake, Elimination and Metabolism of Brominated Dibenzofurans in Mice. Toxics.

[161] Ennour-Idrissi K., Ayotte P., Diorio C. (2019). Persistent Organic Pollutants and Breast Cancer: A Systematic Review and Critical Appraisal of the Literature. Cancers.

[162] Brown L.J., Achinger-Kawecka J., Portman N., Clark S., Stirzaker C., Lim E. (2022). Epigenetic Therapies and Biomarkers in Breast Cancer. Cancers.

[163] Kim A., Mo K., Kwon H., Choe S., Park M., Kwak W., Yoon H. (2023). Epigenetic Regulation in Breast Cancer: Insights on Epidrugs. Epigenomes.

[164] Roy D., Morgan M., Yoo C., Deoraj A., Roy S., Yadav V.K., Garoub M., Assaggaf H., Doke M. (2015). Integrated Bioinformatics, Environmental Epidemiologic and Genomic Approaches to Identify Environmental and Molecular Links Between Endometriosis and Breast Cancer. Int. J. Mol. Sci..

[165] Ganguly S., Arora I., Tollefsbol T.O. (2021). Impact of Stilbenes as Epigenetic Modulators of Breast Cancer Risk and Associated Biomarkers. Int. J. Mol. Sci..

[166] Bhat S.S., Prasad S.K., Shivamallu C., Prasad K.S., Syed A., Reddy P., Cull C.A., Amachawadi R.G. (2021). Genistein: A Potent Anti-Breast Cancer Agent. Curr. Issues Mol. Biol..

[167] Sohel M., Biswas P., Al Amin M., Hossain M.A., Sultana H., Dey D., Aktar S., Setu A., Khan M.S., Paul P. (2022). Genistein, a Potential Phytochemical against Breast Cancer Treatment-Insight into the Molecular Mechanisms. Processes.

[168] Donovan M.G., Selmin O.I., Doetschman T.C., Romagnolo D.F. (2019). Epigenetic Activation of *BRCA1* by Genistein In Vivo and Triple Negative Breast Cancer Cells Linked to Antagonism toward Aryl Hydrocarbon Receptor. Nutrients.

[169] Filippone A., Rossi C., Rossi M.M., Di Micco A., Maggiore C., Forcina L., Natale M., Costantini L., Merendino N., Di Leone A. (2023). Endocrine Disruptors in Food, Estrobolome and Breast Cancer. J. Clin. Med..

[170] Nguyen M., Osipo C. (2022). Targeting Breast Cancer Stem Cells Using Naturally Occurring Phytoestrogens. Int. J. Mol. Sci..

[171] Salmerón-Bárcenas E.G., Zacapala-Gómez A.E., Torres-Rojas F.I., Antonio-Véjar V., Ávila-López P.A., Baños-Hernández C.J., Núñez-Martínez H.N., Dircio-Maldonado R., Martínez-Carrillo D.N., Ortiz-Ortiz J. (2024). TET Enzymes and 5hmC Levels in Carcinogenesis and Progression of Breast Cancer: Potential Therapeutic Targets. Int. J. Mol. Sci..

[172] Zheng K., Lyu Z., Chen J., Chen G. (2024). 5-Hydroxymethylcytosine: Far Beyond the Intermediate of DNA Demethylation. Int. J. Mol. Sci..

[173] Theinel M.H., Nucci M.P., Alves A.H., Dias O.F.M., Mamani J.B., Garrigós M.M., Oliveira F.A., Rego G.N.A., Valle N.M.E., Cianciarullo G. (2023). The Effects of Omega-3 Polyunsaturated Fatty Acids on Breast Cancer as a Preventive Measure or as an Adjunct to Conventional Treatments. Nutrients.

[174] Bobin-Dubigeon C., Nazih H., Croyal M., Bard J.-M. (2022). Link Between Omega 3 Fatty Acids Carried by Lipoproteins and Breast Cancer Severity. Nutrients.

[175] Blewitt M., Whitelaw E. (2013). The Use of Mouse Models to Study Epigenetics. Cold Spring Harb. Perspect. Biol..

[176] Kiyama R. (2023). Estrogenic Flavonoids and Their Molecular Mechanisms of Action. J. Nutr. Biochem..

[177] Pejčić T., Zeković M., Bumbaširević U., Kalaba M., Vovk I., Bensa M., Popović L., Tešić Ž. (2023). The Role of Isoflavones in the Prevention of Breast Cancer and Prostate Cancer. Antioxidants.

[178] Romagnolo D.F., Daniels K.D., Grunwald J.T., Ramos S.A., Propper C.R., Selmin O.I. (2016). Epigenetics of Breast Cancer: Modifying Role of Environmental and Bioactive Food Compounds. Mol. Nutr. Food Res..

[179] Da Cruz R.S., Chen E., Smith M., Bates J., De Assis S. (2020). Diet and Transgenerational Epigenetic Inheritance of Breast Cancer: The Role of the Paternal Germline. Front. Nutr..

[180] Patrizi B., Siciliani de Cumis M. (2018). TCDD Toxicity Mediated by Epigenetic Mechanisms. Int. J. Mol. Sci..

[181] Harandi-Zadeh S., Boycott C., Beetch M., Yang T., Martin B.J.E., Ren K., Kwasniak A., Dupuis J.H., Lubecka K., Yada R.Y. (2021). Pterostilbene Changes Epigenetic Marks at Enhancer Regions of Oncogenes in Breast Cancer Cells. Antioxidants.

[182] Lee R.S., Sad K., Fawwal D.V., Spangle J.M. (2023). Emerging Role of Epigenetic Modifiers in Breast Cancer Pathogenesis and Therapeutic Response. Cancers.

[183] Aldeli N., Murphy D., Hanano A. (2024). Impact of Dioxins on Reproductive Health in Female Mammals. Front. Toxicol..

[184] Hsu C.-N., Hung C.-H., Hou C.-Y., Chang C.-I., Tain Y.-L. (2021). Perinatal Resveratrol Therapy to Dioxin-Exposed Dams Prevents the Programming of Hypertension in Adult Rat Offspring. Antioxidants.

[185] Overhof D.R., Kwekel J.C., Humes D.G., Burgoon L.D., Zacharewski T.R. (2006). Dioxin Induces an Estrogen-Like, Estrogen Receptor-Dependent Gene Expression Response in the Murine Uterus. Mol. Pharmacol..

[186] Ellsworth L., McCaffery H., Chernyak S., Lam S., Sargis R.M., Padmanabhan V., Gregg B. (2020). Lactational Exposure to Polychlorinated Biphenyls Is Higher in Overweight /Obese Women and Associated with Altered Infant Growth Trajectory: A Pilot Study. Curr. Res. Toxicol..

[187] Hang B. (2010). Formation and Repair of Tobacco Carcinogen-Derived Bulky DNA Adducts. J. Nucleic Acids.

[188] White S.S., Fenton S.E., Hines E.P. (2011). Endocrine Disrupting Properties of Perfluorooctanoic Acid. J. Steroid Biochem. Mol. Biol..

[189] Collaborative Group on Hormonal Factors in Breast Cancer (2012). Menarche, Menopause, and Breast Cancer Risk: Individual Participant Meta-Analysis, Including 118 964 Women with Breast Cancer from 117 Epidemiological Studies. Lancet Oncol..

[190] Pierozan P., Jerneren F., Karlsson O. (2018). Perfluorooctanoic Acid (PFOA) Exposure Promotes Proliferation, Migration and Invasion Potential in Human Breast Epithelial Cells. Arch. Toxicol..

[191] Wen Y., Mirji N., Irudayaraj J. (2020). Epigenetic Toxicity of PFOA and GenX in HepG2 Cells and Their Role in Lipid Metabolism. Toxicol. In Vitro.

[192] Qi S.-Y., Xu X.-L., Ma W.-Z., Deng S.-L., Lian Z.-X., Yu K. (2022). Effects of Organochlorine Pesticide Residues in Maternal Body on Infants. Front. Endocrinol..

[193] Aubé M., Larochelle C., Ayotte P. (2008). 1,1-Dichloro-2,2-Bis(p-Chlorophenyl)Ethylene (p,p’-DDE) Disrupts the Estrogen-Androgen Balance Regulating the Growth of Hormone-Dependent Breast Cancer Cells. Breast Cancer Res..

[194] Cocco P., Fadda D., Billai B., D’Atri M., Melis M., Blair A. (2005). Cancer Mortality among Men Occupationally Exposed to Dichlorodiphenyltrichloroethane. Cancer Res..

[195] Strong A.L., Shi Z., Strong M.J., Miller D.F.B., Rusch D.B., Buechlein A.M., Flemington E.K., McLachlan J.A., Nephew K.P., Burow M.E. (2015). Effects of the Endocrine-Disrupting Chemical DDT on Self-Renewal and Differentiation of Human Mesenchymal Stem Cells. Environ. Health Perspect..

[196] Esposito E., Indolfi C., Bello I., Smimmo M., Vellecco V., Schettino A., Montanaro R., Morroni F., Sita G., Graziosi A. (2024). The Endocrine Disruptor Vinclozolin Causes Endothelial Injury via eNOS/Nox4/IRE1α Signaling. Eur. J. Pharmacol..

[197] Van Maele-Fabry G., Lombaert N., Lison D. (2016). Dietary Exposure to Cadmium and Risk of Breast Cancer in Postmenopausal Women: A Systematic Review and Meta-Analysis. Environ. Int..

[198] Pullella K., Kotsopoulos J. (2020). Arsenic Exposure and Breast Cancer Risk: A Re-Evaluation of the Literature. Nutrients.

[199] Peivasteh-roudsari L., Barzegar-bafrouei R., Sharifi K.A., Azimisalim S., Karami M., Abedinzadeh S., Asadinezhad S., Tajdar-oranj B., Mahdavi V., Alizadeh A.M. (2023). Origin, Dietary Exposure, and Toxicity of Endocrine-Disrupting Food Chemical Contaminants: A Comprehensive Review. Heliyon.

[200] Wang Z., Liu H., Liu S. (2017). Low-Dose Bisphenol A Exposure: A Seemingly Instigating Carcinogenic Effect on Breast Cancer. Adv. Sci..

[201] Zuccarello P., Oliveri Conti G., Cavallaro F., Copat C., Cristaldi A., Fiore M., Ferrante M. (2018). Implication of Dietary Phthalates in Breast Cancer. A Systematic Review. Food Chem. Toxicol..

[202] Fiolet T., Casagrande C., Nicolas G., Horvath Z., Frenoy P., Weiderpass E., Katzke V., Kaaks R., Rodriguez-Barranco M., Panico S. (2022). Dietary Intakes of Dioxins and Polychlorobiphenyls (PCBs) and Breast Cancer Risk in 9 European Countries. Environ. Int..

[203] González N., Domingo J.L. (2021). Polychlorinated Dibenzo-p-Dioxins and Dibenzofurans (PCDD/Fs) in Food and Human Dietary Intake: An Update of the Scientific Literature. Food Chem. Toxicol..

